# A tRNA modifying enzyme as a tunable regulatory nexus for bacterial stress responses and virulence

**DOI:** 10.1093/nar/gkac116

**Published:** 2022-02-25

**Authors:** Brittany A Fleming, Matthew G Blango, Alexis A Rousek, William M Kincannon, Alexander Tran, Adam J Lewis, Colin W Russell, Qin Zhou, Lisa M Baird, Amelia E Barber, John R Brannon, Connor J Beebout, Vahe Bandarian, Maria Hadjifrangiskou, Michael T Howard, Matthew A Mulvey

**Affiliations:** Division of Microbiology and Immunology, Pathology Department, University of Utah School of Medicine, Salt Lake City, UT 84112, USA; Junior Research Group RNA Biology of Fungal Infections, Leibniz Institute for Natural Product Research and Infection Biology – Hans Knöll Institute (Leibniz-HKI), 07745 Jena, Germany; Division of Microbiology and Immunology, Pathology Department, University of Utah School of Medicine, Salt Lake City, UT 84112, USA; Department of Chemistry, University of Utah, Salt Lake City, UT 84112, USA; Division of Microbiology and Immunology, Pathology Department, University of Utah School of Medicine, Salt Lake City, UT 84112, USA; Division of Microbiology and Immunology, Pathology Department, University of Utah School of Medicine, Salt Lake City, UT 84112, USA; Division of Microbiology and Immunology, Pathology Department, University of Utah School of Medicine, Salt Lake City, UT 84112, USA; Division of Microbiology and Immunology, Pathology Department, University of Utah School of Medicine, Salt Lake City, UT 84112, USA; Department of Human Genetics, University of Utah, Salt Lake City, UT 84112, USA; Division of Microbiology and Immunology, Pathology Department, University of Utah School of Medicine, Salt Lake City, UT 84112, USA; Department of Pathology, Microbiology, and Immunology, Vanderbilt University Medical Center, Nashville, TN 37232, USA; Department of Pathology, Microbiology, and Immunology, Vanderbilt University Medical Center, Nashville, TN 37232, USA; Department of Chemistry, University of Utah, Salt Lake City, UT 84112, USA; Department of Pathology, Microbiology, and Immunology, Vanderbilt University Medical Center, Nashville, TN 37232, USA; Department of Human Genetics, University of Utah, Salt Lake City, UT 84112, USA; Division of Microbiology and Immunology, Pathology Department, University of Utah School of Medicine, Salt Lake City, UT 84112, USA

## Abstract

Post-transcriptional modifications can impact the stability and functionality of many different classes of RNA molecules and are an especially important aspect of tRNA regulation. It is hypothesized that cells can orchestrate rapid responses to changing environmental conditions by adjusting the specific types and levels of tRNA modifications. We uncovered strong evidence in support of this tRNA global regulation hypothesis by examining effects of the well-conserved tRNA modifying enzyme MiaA in extraintestinal pathogenic *Escherichia coli* (ExPEC), a major cause of urinary tract and bloodstream infections. MiaA mediates the prenylation of adenosine-37 within tRNAs that decode UNN codons, and we found it to be crucial to the fitness and virulence of ExPEC. MiaA levels shifted in response to stress via a post-transcriptional mechanism, resulting in marked changes in the amounts of fully modified MiaA substrates. Both ablation and forced overproduction of MiaA stimulated translational frameshifting and profoundly altered the ExPEC proteome, with variable effects attributable to UNN content, changes in the catalytic activity of MiaA, or availability of metabolic precursors. Cumulatively, these data indicate that balanced input from MiaA is critical for optimizing cellular responses, with MiaA acting much like a rheostat that can be used to realign global protein expression patterns.

## Graphical Abstract

**Graphical Abstract:**
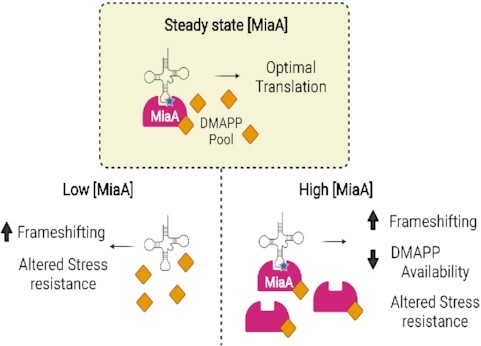
Work in extraintestinal pathogenic *Escherichia coli* indicates that the tRNA-modifying enzyme MiaA can act as a rheostat, with levels shifting in response to environmental cues as a means to modulate bacterial stress resistance and virulence-related phenotypes.

## INTRODUCTION

The translation of mRNA into protein by ribosomes and aminoacyl-transfer RNA (tRNA) complexes is an energy-intensive process that is subject to multiple levels of complicated regulation. For example, tRNAs can be covalently modified by >100 different moieties that can influence the charging of tRNAs with amino acids, tRNA stability, codon usage, and reading frame maintenance (1–4). In *Escherichia coli* and other organisms, the hypomodification of tRNAs can result in decreased growth rates, altered metabolic requirements, and reduced stress resistance (5–8). Shifts in the prevalence of specific tRNA modifications are proposed to help optimize cellular responses to stress by affecting translational fidelity and selective protein expression (9–11). In other words, changing levels of tRNA modifications may control the codon-biased translation of select transcripts, providing a post-transcriptional programmable mechanism that distressed cells can use to facilitate beneficial changes in their proteomes. Although there is growing evidence from a variety of model systems in support of this idea (12,13), our understanding of how changing levels of a tRNA modifying enzyme actually impact the protein landscape is limited.

**Figure 1.**
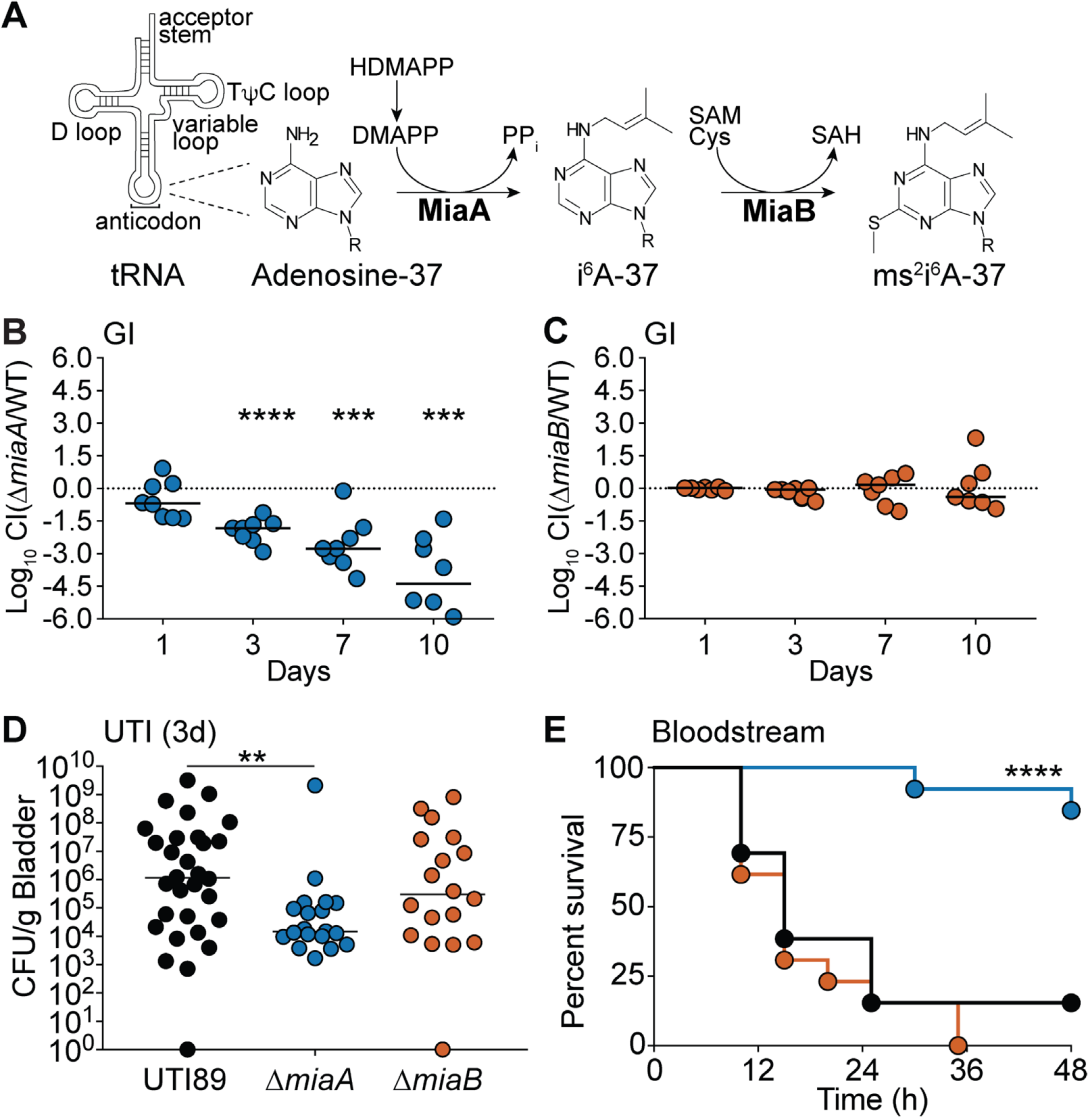
MiaA promotes ExPEC fitness and virulence within diverse host niches. (**A**) MiaA and MiaB act sequentially to modify tRNA molecules that recognize UNN codons; modified from (28). HDMAPP, mono[(2E)-hydroxy-3-methyl-2-butenyl] ester diphosphoric acid; DMAPP, dimethylallyl diphosphate; SAM, S-adenosylmethionine; SAH, S-adenosylhomocysteine; Cys, cysteine. (B and C) To assess gut colonization, adult BALB/c mice were inoculated via oral gavage with ∼10^9^ CFU of a 1:1 mixture of (**B**) UTI89 and UTI89Δ*miaA* or (**C**) UTI89 and UTI89Δ*miaB*. Fecal titers were determined at the indicated time points and used to calculate competitive indices (CI). ***P< 0.001; ****P < 0.0001 by one sample *t*-tests. *n* = 7–8 mice from two independent experiments. (**D**) The bladders of adult female CBA/J mice were inoculated via transurethral catheterization with ∼10^7^ CFU of UTI89, UTI89Δ*miaA*, or UTI89Δ*miaB*. Mice were sacrificed 3 days later and bacterial titers within the bladders were determined by plating tissue homogenates. **P < 0.01 by Mann–Whitney *U* tests; *n*≥ 19 mice per group from at least three independent experiments. In (B), (C) and (D), bars indicate median values; dots represent individual mice. (**E**) Kaplan–Meier survival curves of C57Bl/6 mice inoculated via i.p. injections with ∼10^7^ CFU of UTI89 (black line), UTI89Δ*miaA* (blue) or UTI89Δ*miaB* (orange). ****P < 0.0001 by log-rank Mantel Cox test for UTI89 versus UTI89Δ*miaA*; *n*= 13 mice per group from two independent experiments.

One of the most commonly modified tRNA residues in bacteria is adenosine-37 (A-37), which lies adjacent to the anticodon loop (8,14). In its final form in *E. coli*, A-37 of UNN-recognizing tRNA molecules is oftentimes prenylated and methylthiolated (15). The *miaA* gene of *E. coli* encodes a tRNA prenyltransferase that catalyzes the addition of a prenyl group onto the *N^6^*-nitrogen of A-37 to create i^6^A-37 tRNA (16,17) (Figure 1A). The modified i^6^A-37 residue is subsequently methylthiolated by the radical-S-adenosylmethionine enzyme MiaB to create ms^2^i^6^A-37 (18). The bulky and hydrophobic ms^2^i^6^A-37 modification enhances tRNA interactions with UNN target codons, promoting reading frame maintenance and translational fidelity (5,8,19). Mutations in the *miaA* locus result in an unmodified A-37 residue, as prenylation is required for methylthiolation by MiaB. In laboratory-adapted K-12 *E. coli* strains, mutations in *miaA* impair attenuation of the tryptophan and phenylalanine operons (20,21) and diminish translation of the stationary phase sigma factor RpoS and the small RNA chaperone Hfq (7,22,23). Additionally, mutants lacking *miaA* are unable to effectively resolve aberrant DNA-protein crosslinks (24) and have somewhat elevated spontaneous mutation frequencies (10,25,26). The ms^2^i^6^A-37 modification is highly conserved in both prokaryotes and eukaryotes, though the specific enzymes that mediate this modification have diverged within evolutionarily distant organisms (8). However, in prokaryotes, MiaA and MiaB homologues are relatively well conserved, and the enzymes appear to function similarly in all tested bacterial species (27,28).

Given that the ms^2^i^6^A-37 modification is a well-defined regulator of many tRNA functions in lab-adapted K-12 *E. coli* strains, we sought to understand how this modification is co-opted in a pathogenic *E. coli* background. *E. coli* pathotypes display extensive genetic diversity and are usually much more resilient under stress than their lab-adapted counterparts (29). As such, pathogens can provide insight into stress resistance and adaptive mechanisms that may be difficult to discern in non-pathogenic lab strains. Extraintestinal Pathogenic *E. coli* (ExPEC) typically reside in the lower intestinal tract of mammals, where they are rarely associated with pathology (30). However, when these microbes spread outside the gut to other host sites they can cause a number of serious diseases, including urinary tract and bloodstream infections (29,31). Bacterial pathogens like ExPEC must be able to rapidly respond to a diverse array of stressors encountered within changing host environments. These include nutrient deprivation, oxygen and nitrogen radicals, extremes in pH, envelope damage, changing osmotic pressures, and a wide assortment of host immune effector cells and antimicrobial compounds (32–36). Here, we show that ExPEC can tune MiaA expression in response to stress, and that varying levels of this enzyme can increase translational frameshifting and markedly alter the spectrum of expressed proteins. Our results place MiaA at the center of a regulatory network that can promote stark changes in the proteome via multiple processes, including the alteration of other RNA and translational modifiers and depletion of metabolic precursors.

## MATERIALS AND METHODS

### Study approval

All animals used in this study were handled in accordance with protocols approved by the Institutional Animal Care and Use Committee at the University of Utah (Protocol number 19-01001), following US federal guidelines indicated by the Office of Laboratory Animal Welfare (OLAW) and described in the Guide for the Care and Use of Laboratory Animals, 8th Edition. Mice were purchased from The Jackson Laboratory, housed three to five per cage, and allowed to eat (irradiated Teklad Global Soy Protein-Free Extruded chow) and drink antibiotic-free water *ad libitum*.

### Reagents

Chemicals were procured from Sigma-Aldrich unless otherwise noted. Specialized reagents used for this study included methyl viologen (Sigma-Aldrich), acidified sodium nitrite (Sigma-Aldrich), DMAPP (Dimethylallyl Pyrophosphate; Cayman Chemical), HDMAPP (mono[(2*E*)-hydroxy-3-methyl-2-butenyl] ester diphosphoric acid; Cayman Chemical), and i^6^A (*N*^6^-(Δ^2^-isopentenyl)adenosine; Cayman Chemical).

### Biological resources

Seven- to eight-week-old female C57Bl/6, CBA/J, or C3H/HeJ mice (The Jackson Laboratory) were used according to the experimental descriptions below. Cell culture experiments were performed using the human bladder epithelial cell line 5637 (HTB-9; ATCC) and are described further in the sections below (37). Bacterial strains used in this study are listed in Supplementary Table S1. Mutant strains were constructed in the reference ExPEC isolate UTI89 using the lambda Red recombination system and primers detailed in Supplementary Table S2, as previously described (38). The chloramphenicol resistance (Clm^R^) cassette flanked by LoxP sites was amplified from plasmid pKD3 using primers that contain overhanging ends with ∼40 bp of homology near the 5′ and 3′ ends of each target locus. PCR products were introduced by electroporation into UTI89 carrying pKM208, which encodes an IPTG-inducible lambda Red recombinase (39). Knockouts were verified by PCR using primers indicated in Supplementary Table S2.

Expression and reporter constructs were generated using standard molecular biology approaches and primers listed in Supplementary Table S2. The *miaA* and *miaB* genes were amplified from UTI89 by PCR, digested, and ligated into pRR48 using Pst1 and Kpn1 restriction sites to create pMiaA_P_*_tac_* and pMiaB_P_*_tac_*. Sequences encoding Hfq fused with C-terminal Flag and 6xHis tags were cloned using a similar approach to make pHfq_P_*_tac_*. Point mutations in pMiaA-Flag_P_*_tac_* were introduced using the QuikChange II site-directed mutagenesis kit (Agilent) with the primers noted in Supplementary Table S2. To create pMiaA_nat_, the UTI89 *miaA* coding sequence was amplified along with 200 bp of flanking sequences, including the *miaA* promoter, and then ligated into the EcoR1 site of pACYC184. The plasmid pMiaA-Flag_nat_, having the *miaA* promoter region upstream of sequences encoding MiaA with a C-terminal Flag-tag, was produced similarly. To create pMiaA-Flag_P_*_tac_*, sequences for Flag-tagged MiaA were sub-cloned from pMiaA_nat_ into the PstI and KpnI sites of pRR48. Of note, the Flag tag did not interfere with MiaA function in complementation assays.

The dual-luciferase reporter plasmids used for frameshifting assays were created using p2Luc plasmids as templates (40,41). The genes encoding the renilla and firefly luciferases were amplified by PCR along with intergenic Az1- or HIV-derived linker sequences. A Shine-Dalgarno ribosome binding site was incorporated into the forward primer (p2Luc_F) to promote translation of the linked luciferases. PCR products were digested and ligated into the KpnI and HindIII sites of pBAD18 (Ap^R^) and pBAD33 (Cam^R^). Plasmids with different resistance cassettes were needed for use with UTI89Δ*miaA* (Cam^R^) and UTI89 carrying pMiaA_P_*_tac_* or the empty vector pRR48. Control plasmids in which the Az1 and HIV linkers are altered to place the two luciferases in-frame were generated in an analogous fashion using previously described p2Luc plasmids as templates (40,41).

### Bacterial growth analysis

UTI89 and its derivatives were grown from frozen stocks in 5 ml of LB (lysogeny broth), 100 mM MES-buffered LB (MES-LB; pH 5.0), or modified M9 medium (6 g/liter Na_2_HPO_4_, 3 g/l KH_2_PO_4_, 1 g/l NH_4_Cl, 0.5 g/l NaCl, 1 mM MgSO_4_, 0.1 mM CaCl_2_, 0.1% glucose, 0.0025% nicotinic acid, 0.2% casein amino acids and 16.5 μg/ml thiamine in H_2_O) at 37°C overnight in loosely capped 20-by-150-mm borosilicate glass tubes with shaking (225 rpm, with tubes tilted at a 30° angle). Overnight cultures were brought to an OD_600_ of ∼1.0 and then sub-cultured 1:100 into LB, MES-LB or M9 medium. Growth curves were acquired using a Bioscreen C instrument (Growth Curves USA) with 200-μl cultures in 100-well honeycomb plates shaking at 37°C. Cultures included extra NaCl (5% w/v), 1 mM MV (Sigma-Aldrich), 1 or 2 mM ASN (Sigma-Aldrich), 25 or 50 μM HDMAPP or DMAPP (Cayman Chemical), or IPTG, as indicated. Solutions containing MV, ASN, HDMAPP, or DMAPP were prepared fresh just before use. All growth curves were determined using quadruplicate samples with at least three independent replicates. Overnight cultures of strains carrying plasmids for complementation experiments were grown in the presence of antibiotics (100 μg of ampicillin/ml or 50 μg of tetracycline/ml) to maintain the plasmids, but antibiotics were not included in media used for the subsequent growth assays. Error bars are small for technical replicates in these experiments and were omitted for clarity. As a means to show levels of variance between each replicate, graphs with area under the curve (AUC) values for all growth curves are presented in Supplementary Figure S1.

### Invasion, adhesion, and intracellular persistence assays

Bacteria were grown at 37°C for 48 h in 20 ml static LB to induce expression of type 1 pili, which are important mediators of UPEC adherence and entry into host cells (42). Host cell association and gentamicin protection-based invasion and overnight intracellular persistence assays were performed as previously described using the human bladder epithelial cell line 5637 (HTB-9; ATCC) (37). Of note, UTI89Δ*miaA* is about 3-fold more sensitive to the host-cell impermeable antibiotic gentamicin, as determined by using Etest Strips (VWR) (Supplementary Figure S2). However, this likely had no effect on results from the cell culture-based invasion and intracellular survival experiments, as the concentrations of gentamicin (100 and 10 μg/ml) used in these assays exceed those needed to effectively kill extracellular UTI89, UTI89Δ*miaA*, and UTI89Δ*miaB*.

### Competitive gut colonization assays

For these assays, a kanamycin resistance cassette (Kan^R^) was inserted into the *att*Tn7 site of UTI89 to create UTI89::Kan^R^, which can be easily distinguished from the chloramphenicol resistant (Cam^R^) *miaA* and *miaB* knockout mutants by plating on selective media. Previous work demonstrated that insertion of the Kan^R^ resistance cassette into the *att*Tn7 site does not impact ExPEC fitness within the gut (43,44). Individual cultures of UTI89::Kan^R^ (standing in as the wild-type strain), UTI89Δ*miaA*, and UTI89Δ*miaB* were grown statically from frozen stocks for 24 h at 37°C in 250-ml flasks containing 20 ml of modified M9 medium. Each knockout mutant was then mixed 1:1 with UTI89::Kan^R^ (6 ml of each culture) and then pelleted by centrifugation at 8000 × *g* for 8 min at room temperature. The bacterial pellets were then washed once with phosphate-buffered saline (PBS), pelleted again, and resuspended in 0.5 ml of PBS. Female SPF BALB/c mice aged 7 to 8 weeks were inoculated via oral gavage with 50 μl PBS containing ∼10^9^ CFU of each bacterial mixture. At the indicated time points post-inoculation, individual mice were placed into unused takeout boxes for a few minutes for weighing and feces collection. Freshly deposited feces were collected from the boxes and immediately added to 1 ml of 0.7% NaCl, weighed, and set on ice. The samples were then homogenized and briefly centrifuged at low speed to pellet any insoluble debris. Supernatants were serially diluted and plated onto LB agar containing either chloramphenicol (20 μg/ml) or kanamycin (50 μg/ml) for selective growth of UTI89::Kan^R^ (wild type), UTI89Δ*miaA*, or UTI89Δ*miaB*. Fecal samples were also analyzed prior to the start of each experiment to ensure that there were no endogenous bacteria present that were resistant to chloramphenicol or kanamycin. Competitive indices (CI) were calculated as the ratio of knockout over wild-type bacteria recovered in the feces divided by the ratio of knockout over wild-type bacteria present in the inoculum (44,45). A total of seven to eight mice in two independent assays were used for each set of bacterial strains tested.

### UTI model

The murine UTI model was used essentially as described by our group and others (42,46). Wild-type UTI89 and the *miaA*, and *miaB* knockout mutants were grown from frozen stocks in 20 ml LB in 250 ml Erlenmeyer flasks without shaking at 37°C for 24 h. Bacteria were then pelleted by centrifugation (8 min at 8000 × g) and resuspended in PBS. Seven- to eight-week-old female CBA/J or C3H/HeJ mice were briefly anesthetized by isoflurane inhalation and slowly inoculated via transurethral catheterization with 50 μl of PBS containing a suspension of ∼10^7^ bacteria. Bacterial reflux into the kidneys using this procedure is rare, occurring in less than 1% of the test animals. At 0.25, 1, 3 or 9 days post-inoculation, mice were sacrificed and bladders were harvested aseptically, weighed and homogenized in 1 ml PBS containing 0.025% Triton X-100. Bacterial titers within the homogenates were determined by plating serial dilutions on LB agar plates. Nine or more mice in total, from two independent experiments, were used for each bacterial strain and time point examined.

### Sepsis model

UTI89, UTI89Δ*miaA*, and UTI89Δ*miaB* were grown from frozen stocks in 20 ml M9 broth without shaking at 37°C for 24 h, pelleted by centrifugation at 8000 × *g* for 8 min, and washed once with PBS, pelleted again, and resuspended in PBS. Seven- to eight-week-old female C57Bl/6 mice were briefly anesthetized by isoflurane inhalation and infected via intraperitoneal injection of ∼10^7^ CFU within 200 ml PBS. Mice were monitored over a 72-h period for signs of morbidity and mortality. Alternatively, at 6 h post-inoculation mice were sacrificed and the liver, kidneys, and spleens were harvested aseptically, weighed, and homogenized in 1 ml PBS containing 0.025% Triton X-100. Bacterial titers within the homogenates were determined by plating serial dilutions on LB agar plates.

### Biofilm analysis

*In vitro* rugose biofilm assays were performed starting with cultures grown overnight at 37°C shaking in LB, as described (47). Bacteria from each culture were then brought to an OD_600_ of ∼1.0 and 10 μl aliquots were spotted onto YESCA agar plates (12 g/l Casamino acids, 1.2 g yeast extract, 22 g agar) and incubated at RT (∼20–22°C). After 14 days, biofilm images were acquired by focus stacking using an M.Zuiko Digital ED 60 mm lens mounted on an Olympus OM-D E-M1 Mark II camera.

### Motility assays

Cultures of UTI89, UTI89Δ*miaA*, and UTI89Δ*miaB* grown overnight shaking in LB or M9 medium were brought to OD_600_ of 1.0. Swim motility plates, containing 0.2% agar in LB or M9 medium, were inoculated with 2 μl of each bacterial suspension delivered just below the agar surface. The diameter of bacterial spreading was measured every 1–2 h over the course of an 8–10 h-incubation at 37°C. Swim rates were calculated during logarithmic growth. To assess the effects of MiaA and MiaB overexpression on motility, tryptone soft agar plates (48) containing 50 μg/ml ampicillin and 100 μM IPTG were inoculated with UTI89/pRR48, UTI89/pMiaA_P_*tac* and UTI89/pMiaB_P_*_tac_* from overnight shaking cultures. Plates were imaged after a 6-h incubation at 37°C.

### Acid resistance assays

Bacterial strains from overnight cultures were diluted 1:100 in fresh LB and grown shaking at 37°C for 3 h. Concentrated HCl was then added to each culture to adjust the pH to 3.0 and incubations were continued for another 30 min. Bacteria from 1 ml of each culture were then pelleted at 16 000 × *g* for 5 min and washed in PBS. Surviving bacteria were enumerated by plating serial dilutions on LB agar and normalized to input titers.

### Osmotic stress resistance assays

UTI89/pACYC184, UTI89Δ*miaA*/pACYC184, UTI89Δ*miaA*/pMiaA_nat_, and UTI89Δ*miaB* were grown shaking overnight at 37°C in 5 ml LB broth with 20 μg/ml tetracycline and then back diluted 1:100 into 5 ml fresh LB (+ tetracycline). After 5 h shaking at 37°C, a 1-ml aliquot of each culture was pelleted, resuspended in 1 ml of sterile water with or without 0.1% glucose, and incubations were continued for another 2 h with shaking at 37°C. Viable bacteria present at 0, 30, 60, 90, and 120 min after resuspension in water were quantified by dilution plating and normalized to input titers. Growth curves in LB ± 5% NaCl were acquired as described above.

### Western blot analysis

Bacterial pellets were frozen at −80°C and then resuspended in B-PER lysis reagent (Thermo Scientific) supplemented with 1 mM phenylmethylsulfonyl fluoride, protease inhibitor cocktail (Roche), and Lysonase Bioprocessing Reagent (Novagen). After a 15-min incubation at room temperature, samples were spun for 1 min at 13000 × *g* to remove large cell debris, and protein concentrations in the supernatants were determined using the BCA reagent system (Pierce). Equivalent protein amounts were resolved by SDS-PAGE and subsequently transferred to Immobilon PVDF-FL membranes (Millipore). Blots were probed using mouse anti-Flag M2 (1:3000; Sigma-Aldrich), rabbit anti-Flag (Immunology Consultants Laboratory, inc.), mouse anti-RpoS (anti-SigmaS; Biolegend), or rabbit anti-*E. coli* antisera (1:2000 or 1:5000; BioDesign International) and visualized using enhanced chemiluminescence with HRP-conjugated secondary antibodies (1:3000 or 1:5000; Amersham Biosciences), as described (49). Alternatively, blots were visualized using IRDye-labeled secondary antibodies (1:20 000) and an Odyssey Infrared Imaging System (LI-COR Biosciences).

### RT-qPCR analysis

UTI89 was diluted 1:100 from overnight cultures into fresh LB, grown shaking for 2.5 h at 37°C prior to resuspension in LB or LB + 5% NaCl. After another 1-h incubation bacteria were pelleted and total RNA was extracted using the miRNeasy mini kit (QIAGEN). RNA samples were treated with RNase-Free DNase (QIAGEN) and cDNA was made using SuperScript IV VILO Master Mix (Invitrogen) according to the manufacturer's protocol. Quantitative PCR (qPCR) was carried out using primers listed in Supplementary Table S2 with the PowerUp SYBR Green Master Mix (Thermo Fisher Scientific) on a QuantStudio 3 Real-Time PCR Instrument (Applied Biosystems). Replicas were made for each cDNA sample and *miaA* and *miaB* levels were normalized to *rpoD*, which is known to be stably expressed across a variety of growth stages and conditions (50,51). Products were resolved in 1.5% agarose gels, stained with ethidium bromide, and visualized using a GelDoc system (BioRad Technologies) to help verify the specificity of the RT-qPCR results.

### Frameshifting quantification

UTI89, UTI89Δ*miaA*, UTI89/pRR48, and UTI89/pMiaA_P_*_tac_* carrying one of the dual-luciferase reporter plasmids (see Supplementary Table S1) were grown overnight in LB supplemented with chloramphenicol (20 μg/ml) or ampicillin (100 μg/ml). The cells were sub-cultured 1:100 into 6 ml LB with and without 1 mM IPTG. At an OD_600_ of 0.2, arabinose (0.2%) was added to all of the cultures to induce expression of the luciferases. Cells were allowed to continue growing until reaching an OD_600_ of 0.5, at which point the cultures were adjusted to an OD_600_ of 1.0 and pelleted by spinning at 8000 × *g* for 1.5 min. The pellets were then subjected to one freeze-thaw cycle before being resuspended in Passive Lysis Buffer (Promega; E1910). One scoop of 0.15 mm zirconium oxide beads (Next Advance; ZrOB015) was added to each tube and bacteria were lysed using a Bullet Blender (Next Advance) set at speed 8 for 3 min. After a 30-s spin in a microfuge to pellet beads and any large debris, the supernatants were collected, and luciferase activities were analyzed as previously described (40). Briefly, the Dual-Luciferase Reporter Assay System (Promega) was used in combination with the Veritas Microplate Luminometer from Turner Biosystems to quantify activities of the two luciferases. Frameshifting was calculated by first determining the ratio of firefly to renilla luciferase activity for each sample, and then normalizing each out-of-frame construct (pCWR43 and pCWR45) with their associated in-frame control (pCWR42 and pCWR44, respectively).

### Analysis of relative i^6^A and ms^2^i^6^A levels

UTI89 and UTI89Δ*miaA* were grown from frozen stocks shaking at 37°C overnight in LB. UTI89/pRR48 and UTI89/pMiaA_Ptac_ were grown similarly using LB supplemented with ampicillin (100 μg/ml). The bacteria were sub-cultured 1:100 into 6 ml of LB ± 1 mM IPTG and then grown shaking to an OD_600_ of 0.5. After adjusting the cultures to OD_600_ of 1.0, the bacteria were pelleted by spinning at 8000 × g for 1.5 min. Pellets were then resuspended in 1 ml of RNAlater Stabilization Solution (ThermoFisher) and stored at 4°C overnight prior to extraction of RNA using a Norgen Total RNA Extraction Kit.

RNA samples were analyzed using a Hypersil GOLD C18 column (2.1 mm × 150 mm, 1.9 μm particle size; Thermo Fisher) attached to a Thermo Scientific Dionex UltiMate 3000 UHPLC instrument in line with an LTQ-OrbiTrap XL instrument (Thermo Fisher). The LC-MS parameters were based upon a procedure described previously (52,53), with the following adjustments. The UHPLC column was pre-equilibrated in 100% Buffer A [50 mM ammonium acetate (Fisher) in LC–MS Optima water]. Buffer B consisted of 60% (v/v) LC–MS Optima acetonitrile (Fisher) and 40% LC–MS water (Fisher). The reaction components were eluted at a rate of 0.2 ml/min with the following program: 0% B from 0 to 3.46 min, 0 to 0.9% B from 3.46 to 3.69 min, 0.9 to 1.5% B from 3.69 to 3.92 min, 1.5 to 3% B from 3.92 to 4.25 min, 3 to 20% B from 4.25 to 6.5 min, 20 to 25% B from 6.5 to 7 min, 25 to 40% B from 7 to 8.5 min, 40 to 45% B from 8.5 to 9.25 min, 45 to 60% B from 9.25 to 9.95 min, 60 to 100% B from 9.95 to 10.45 min, 100% B from 10.45 to 16 min, 100 to 0% B from 16 to 16.1 min, and 0% B from 16.1 to 20 min. The flow from the column was diverted to the mass spectrometer from 3.5 to 17 min during the UHPLC program. The mass spectrometer was operated in positive ion mode, and authentic guanosine material (Sigma-Aldrich) was used to generate a tune file for the instrument. The observed *m*/*z* values of the +1 charge states of the i6a and ms2i6a RNA bases were 336.1658 and 382.1535, respectively. The observed retention times for i^6^A and ms^2^i^6^A were determined from the center of their extracted ion chromatogram peaks to be 15.55 and 16.45 min respectively. The retention time of i^6^A from biological extracts was consistent with the retention time of authentic i^6^A material (Cayman Chemical). Absolute intensities of the i^6^A ions were retrieved from the mass spectrum scanned between 15.4 and 16.1 min, while the absolute intensities of the ms^2^i^6^A ions were retrieved from the mass spectrum scanned between 16.2 and 17.0 min. The scan range was chosen to include the entire peak of an EIC trace, excluding mass spectral data recorded out of these bounds. This ensured that the intensities of ions 336.17 and 382.15 arise from eluted i^6^A and ms^2^i^6^A material and did not include background during the rest of the run. The scan windows were wide enough to account for any small drift in retention that might occur from sample to sample. Because total RNA concentration varied by sample, the samples were normalized against the total RNA concentration of each sample, as estimated via NanoDrop measurements at 260 nm.

### Proteomics

The ExPEC isolate UTI89 and its derivatives UTI89Δ*miaA*, UTI89/pRR48, and UTI89/pMiaA_P_*_tac_* were grown from frozen stocks in 5 ml of LB at 37°C overnight in loosely capped 20-by-150-mm borosilicate glass tubes with shaking (225 rpm, with tubes tilted at a 30° angle). UTI89/pRR48 and UTI89/pMiaA_P_*_tac_* were grown overnight in the presence of ampicillin (100 μg/ml) to help maintain the plasmids, but the antibiotic was not included in subsequent steps. Overnight cultures were brought to an OD_600_ of ∼1.0 and then sub-cultured 1:100 into LB and grown to mid-log phase (OD_600_ ∼ 0.5) in LB shaking at 37°C. IPTG (1 mM) was included for UTI89/pRR48 and UTI89/pMiaA_P_*_tac_*. About 1 × 10^9^ CFU from each culture was pelleted at 8000 × *g* for 1.5 min. Supernatants were then removed, and cells from three independent replicates were plunged into liquid nitrogen. Shotgun proteomic analysis of cell lysates was performed with the MSRC Proteomics Core at Vanderbilt University by first partially resolving 20 μg of protein lysates about 1.5 cm using a 10% Novex precast gel, excising the protein region, and then performing in-gel tryptic digestion to recover peptides. These peptides were analyzed by high-resolution data dependent LC–MS/MS. Briefly, samples were autosampled onto a 200 mm by 0.1 mm (Jupiter 3 micron, 300A) self-packed analytical column coupled directly to a Q-exactive plus mass spectrometer (ThermoFisher) using a nanoelectrospray source and resolved using an aqueous to organic gradient. Both the intact masses (MS) and fragmentation patterns (MS/MS) of the peptides were collected in a data-dependent manner utilizing dynamic exclusion to maximize depth of coverage. Resulting peptide MS/MS spectral data were searched against the UTI89 protein database using MaxQuant-LFQ along with subsequent MS1-based integrations and normalizations (54).

Label-free quantification (LFQ) values were loaded into Prostar software for statistical analysis and visualization. The data set was filtered by requiring all conditions to contain at least two values. Imputation for partially observed values was done with the Structured Least Square Adaptative algorithm. Imputation for conditions in which values were missing for a specific protein in all three biological replicates used the DetQuantile algorithm with the settings Quantile:2.5 and Factor:1. Statistical analysis was performed using the 1vs1 settings and Student's *t*-tests. Differentially expressed proteins were categorized (Supplementary Dataset S1) based on literature searches and information drawn from EcoCyc ((55); http://ecocyc.org/), STRING Protein–Protein Interaction Networks Functional Enrichment Analysis ((56); https://string-db.org/), and Phyre2 ((57); http://www.sbg.bio.ic.ac.uk/∼phyre2/html/page.cgi?id=index).

### Statistical analyses

The proteomics data were analyzed as described above. Two-sided Spearman's rank correlation coefficients (rho) and FDR-corrected *P* values were calculated using R version 4.0.5. For all other data, *P* values were determined by Log-Rank (Mantel–Cox), Mann–Whitney U tests, ANOVA, or two-tailed Student's *t*-tests performed using Prism 9.0.0 software, with corrections as indicated (GraphPad Software). AUC values for growth curves were also calculated and graphed using Prism software. Data distribution normality (Gaussian) was not assumed, such that non-parametric tests were used for the mouse experiments. *P* values of less than or equal to 0.05 were defined as significant.

## RESULTS

### MiaA promotes ExPEC fitness and virulence *in vivo*

To assess the importance of MiaA and MiaB for ExPEC within varied host environments, we employed well-established mouse models of gut colonization, urinary tract infection (UTI), and bloodstream infection (58). For these and subsequent experiments, *miaA* and *miaB* were independently deleted from the ExPEC reference strain UTI89 to generate the isogenic knockout mutants UTI89Δ*miaA* and UTI89Δ*miaB* (38,39).

#### Gut colonization

The mammalian gastrointestinal (GI) tract serves as a major reservoir for ExPEC that can seed extraintestinal infections (59–63). Roles for MiaA and MiaB in ExPEC colonization of the GI tract were probed using competitive assays in which ∼10^9^ colony forming units (CFU) of a 1:1 mixture of UTI89 and either UTI89Δ*miaA* or UTI89Δ*miaB* were introduced into adult specific-pathogen-free (SPF) BALB/c mice via intragastric gavage (43–45). In this model system, the levels of ExPEC recovered from the feces reflect ExPEC titers within the large intestines (44). For these assays, UTI89 and the *miaA* and *miaB* knockout mutants were engineered to express either kanamycin (Kan^R^) or chloramphenicol (Cam^R^) resistance cassettes so that the strains could be readily identified by plating fecal homogenates on selective media. Feces were collected at the indicated time points and the numbers of viable bacteria were enumerated to determine competitive indices (CI). UTI89Δ*miaA* was significantly outcompeted by wild-type UTI89 as early as day 3 post-inoculation (Figure 1B). By day 10, there was about a 25 000-fold reduction in the relative levels of UTI89Δ*miaA* recovered from the feces, correlating with a median CI of –4.39. At this time point, UTI89Δ*miaA* titers in the majority of mice were below the limit of detection. In contrast, there were no notable differences in titers between UTI89Δ*miaB* and UTI89 in the feces at any time point (Figure 1C). These results indicate that the loss of MiaA, but not MiaB, greatly impairs the fitness of UTI89 within the gut.

#### UTI

During the course of a UTI, ExPEC is able to bind and invade the host epithelial cells that comprise the bladder mucosa (64). Once internalized into bladder cells, ExPEC can traffic into late endosome-like compartments where it may form quiescent reservoir populations that promote long-term bacterial persistence. Alternatively, ExPEC can enter the host cytosol and rapidly multiply, forming large intracellular bacterial communities that eventually rupture the epithelial cell. In cell culture-based assays using a bladder epithelial cell line, we found that UTI89Δ*miaA* and UTI89Δ*miaB* are able to bind, invade, and survive intracellularly in overnight assays much like wild-type UTI89 (Supplementary Figure S3).

To investigate MiaA and MiaB requirements during UTI, 10^7^ CFU of wild-type UTI89, UTI89Δ*miaA*, and UTI89Δ*miaB* were independently inoculated via transurethral catheterization into adult female SPF CBA/J mice and bacterial titers in the bladders were determined after 3 days. In this analysis, UTI89Δ*miaB* showed no statistically significant defect relative to the parent strain, whereas the Δ*miaA* strain was clearly attenuated (Figure 1D). Deficiencies in bladder colonization by UTI89Δ*miaA* were apparent by 6 h post-inoculation (Supplementary Figure S4A) and were still significant after 9 days (Supplementary Figure S4B). The differences observed between wild-type UTI89 and UTI89Δ*miaA* at 3 days post-inoculation of CBA/J mice were also manifest in C3H/HeJ mice (Supplementary Figure S4C). Due to defects in Toll-like receptor 4 (TLR4) signaling and other innate defenses, C3H/HeJ mice have attenuated inflammatory responses and increased susceptibility to UTI (65–68). Our results indicate that the decreased capacity of UTI89Δ*miaA* to colonize the bladder is not attributable to an inability of the *miaA* knockout to handle TLR4-dependent innate host defenses. Collectively, these results indicate that MiaA is required for maximal fitness in mouse UTI models, while MiaB is less critical.

#### Bloodstream infection

ExPEC is a leading cause of bloodstream infections, which too often trigger discordant systemic inflammatory responses that can result in a life-threatening condition known as sepsis (69). To examine the contributions of MiaA and MiaB to ExPEC virulence and fitness in a model of sepsis, adult SPF C57Bl/6 mice were inoculated via intraperitoneal (i.p.) injections with ∼10^7^ CFU of wild-type UTI89, UTI89Δ*miaA*, or UTI89Δ*miaB*. Following i.p. injection, the bacteria enter the bloodstream and disseminate (43,70,71). In our experiments, only 15% (2/13) of the mice infected with wild-type UTI89, and 0% (0/13) of the mice injected with UTI89Δ*miaB*, were viable after 48 h (Figure 1E). In sharp contrast, 84% (11/13) of the mice infected with UTI89Δ*miaA* survived. At 6 h post-injection, significantly lower numbers of bacteria were recovered from the spleens and kidneys of UTI89Δ*miaA*-infected mice, relative to mice infected with wild-type UTI89 or UTI89Δ*miaB* (Supplementary Figure S5A and B). While not significant, titers in the liver also trended lower in UTI89Δ*miaA*-infected mice (Supplementary Figure S5C). Combined, these data demonstrate that MiaA is important for the virulence of ExPEC and its survival during systemic infections, while MiaB appears dispensable.

### MiaA enhances ExPEC growth and stress resistance

**Figure 2.**
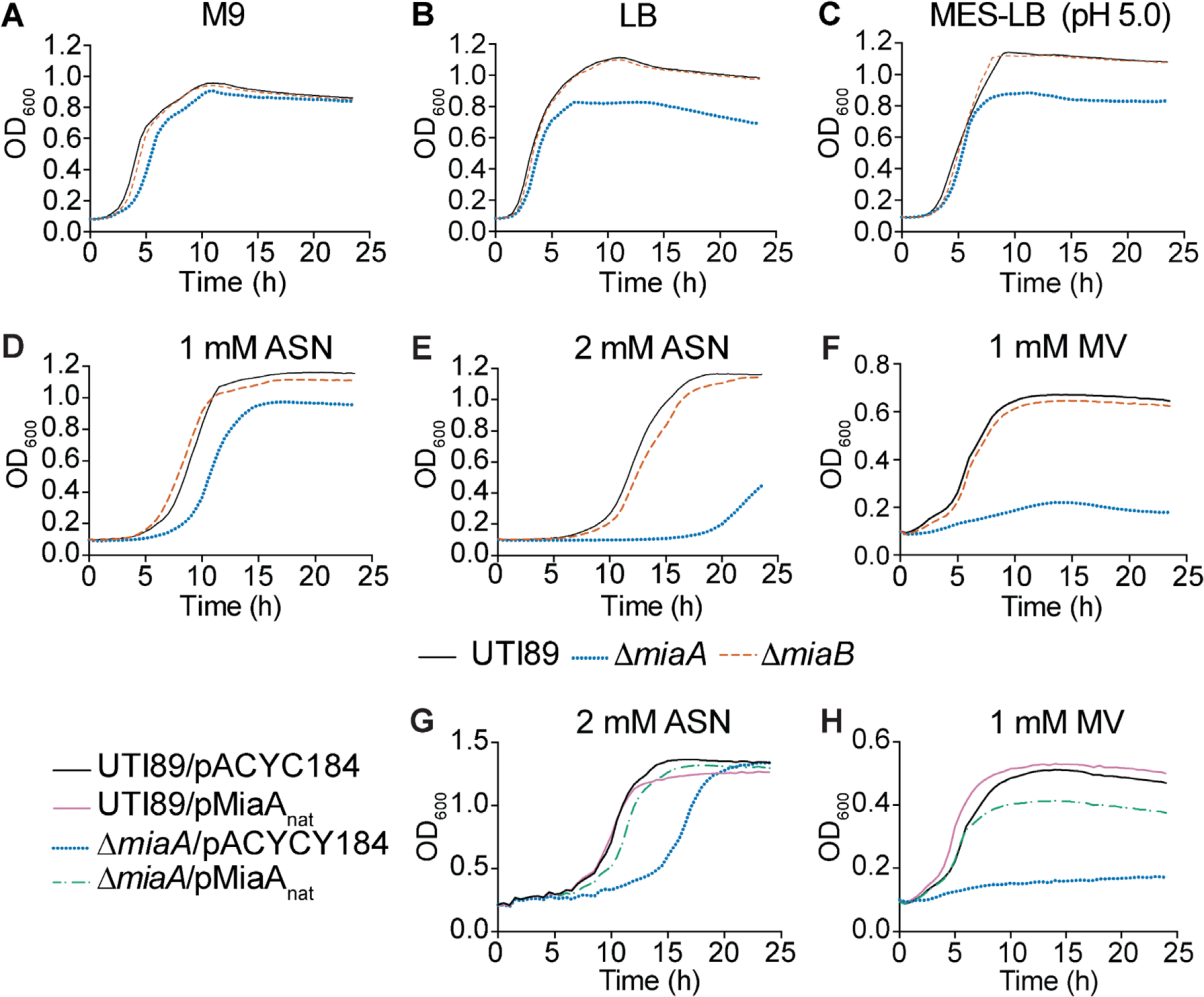
Deletion of *miaA* limits growth of UTI89 in rich medium and lowers resistance to oxidative and nitrosative stress. (**A-F**) Graphs indicate mean growth of UTI89, UTI89Δ*miaA*, and UTI89Δ*miaB* in shaking cultures with modified M9 media, LB, MES-LB, MES-LB with 1 or 2 mM ASN, or LB with 1 mM MV. (**G** and **H**) Curves show mean growth of UTI89 and UTI89Δ*miaA* carrying pMiaA_nat_ (with *miaA* expressed from its native promoter) or the empty vector control pACYC184 in LB containing (**G**) 2 mM ASN or (**H**) 1 mM MV. Each curve shows the means of results from four replicates and are representative of three independent experiments.

Earlier studies showed that K-12 *E. coli* and *Salmonella* mutants lacking *miaA* are moderately impaired in nutrient-rich broth, but less so in nutrient-limited media (19,26,72). Using *in vitro* growth assays, we found that UTI89Δ*miaA* grew normally in modified M9 minimal media but failed to reach densities as high as the wild-type strain in more complex, nutrient-rich lysogeny broth (LB) (Figure 2A and B). In contrast, the *miaB* knockout exhibited no overt growth defects in either type of media. These data suggest that UTI89Δ*miaA* has reduced metabolic flexibility relative to wild-type UTI89 and the *miaB* mutant. This may contribute to the decreased fitness of UTI89Δ*miaA* in our mouse models, where the bacteria likely encounter marked shifts in nutrient availability (58,73,74). However, within different host environments ExPEC will face a wide variety of additional challenges that might be countered by MiaA-dependent processes. We investigated this possibility by examining the effects of MiaA and MiaB on ExPEC resistance to nitrosative, oxidative, and osmotic stress.

#### Oxidative and nitrosative stress

During the course of an infection, both host and bacterial cells can produce reactive oxygen and nitrogen radicals that can damage lipids, proteins, and nucleic acids (75,76). The contributions of MiaA and MiaB to nitrosative and oxidative stress resistance were assessed using acidified sodium nitrite (ASN) and methyl viologen (MV), respectively. When added to low pH morpholineethanesulfonic acid (MES)-buffered LB (MES-LB; pH 5.0), sodium nitrite dismutates to form nitrous acid which in turn generates NO and other harmful reactive nitrogen species (77). In un-supplemented MES-LB, UTI89Δ*miaA* reached a lower maximal density than wild-type UTI89 (Figure 2C), similar to results obtained using standard LB (Figure 2B). The addition of 1 mM ASN delayed entry of UTI89Δ*miaA* into exponential growth phase by close to 4 h (Figure 2D), while 2 mM ASN delayed growth by more than 15 h relative to wild-type UTI89 (Figure 2E). The addition of 1 mM MV, which produces superoxide radicals (78), had even stronger inhibitory effects on growth of UTI89Δ*miaA* (Figure 2F). In contrast, UTI89Δ*miaB* grew much like the wild-type strain in the presence of ASN or MV (Figure 2D–F). Complementation with pMiaA_nat_, a low copy plasmid that encodes MiaA under control of its native promoter, restored growth of UTI89Δ*miaA* to near wild-type levels in both 2 mM ASN (Figure 2G) and in 1 mM MV (Figure 2H).

#### Osmotic stress

**Figure 3.**
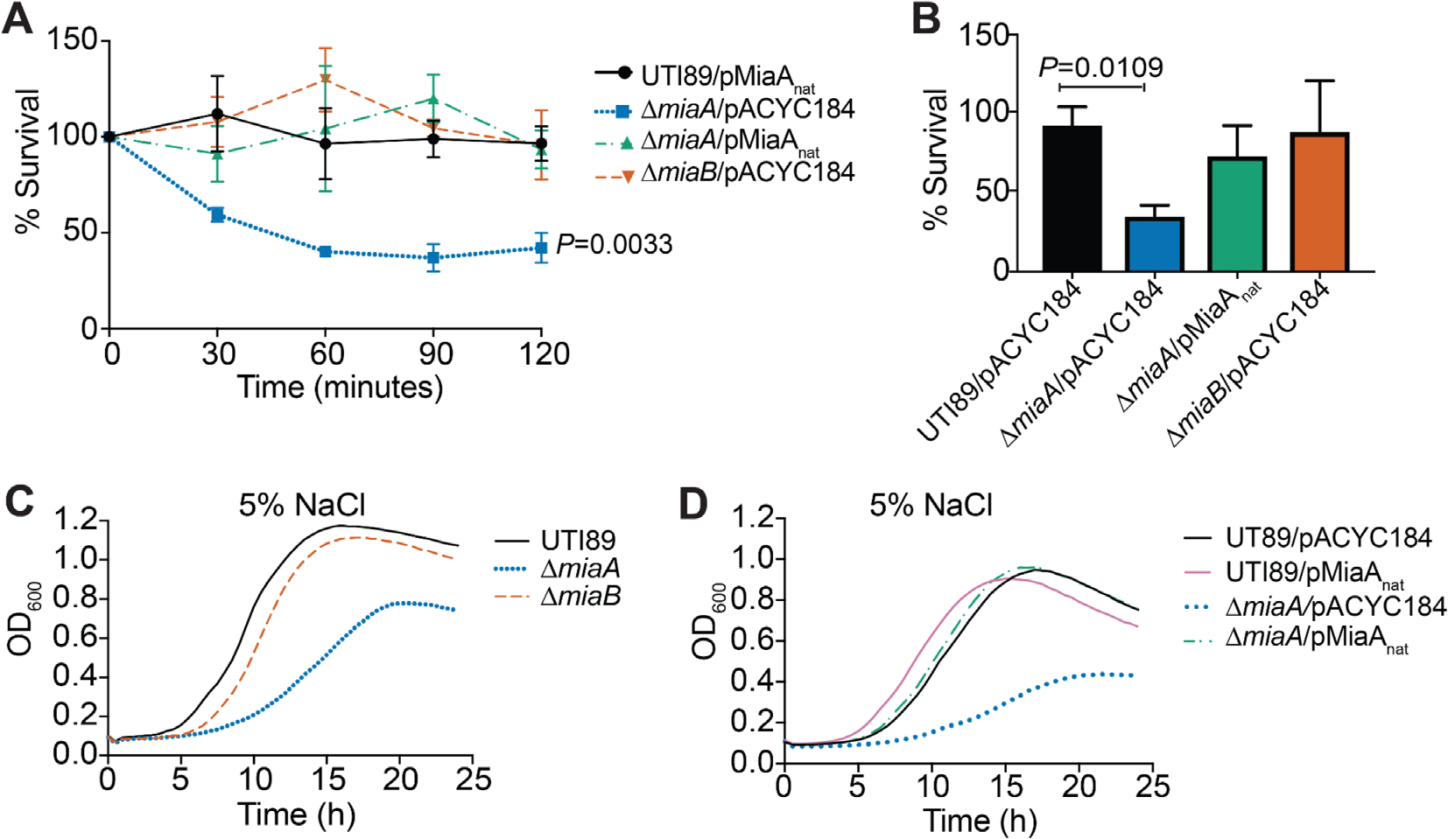
MiaA enhances the resistance of UTI89 to osmotic stress. (**A**) Bacteria were grown to stationary phase in LB, pelleted, and resuspended in ddH_2_O. The mean numbers (±SEM) of surviving bacteria recovered at the indicated time points are graphed as the percentage of the bacteria present immediately after resuspension in ddH_2_O (time 0). *P* values were determined by two-way ANOVA with the Geisser-Greenhouse correction; *n* = 3 independent assays each done in triplicate. (**B**) Bars indicate mean numbers of bacteria (±SD) that survived 2 h in ddH_2_O with 0.1% glucose, calculated as a percent of the inoculum. *P* values were determined, relative to the control strain UTI89/pMiaA_nat_, by unpaired *t*-tests with Welch's correction; *n* = 3 independent assays. (**C** and **D**) Curves show growth of the indicated strains in LB plus 5% NaCl, as measured by OD_600_. Each curve indicates averaged results from quadruplicate replicates and are representative of three independent experiments.

During a UTI, osmotic pressure within the bladder can shift from 50 to >1400 mOsm/kg due to varying concentrations of solutes like sodium and urea (79,80). By comparison, the normal osmolarity of blood ranges from about 275 to 295 mOsm/kg. To test the sensitivities of wild-type UTI89 and the knockout strains to hypoosmotic stress, we diluted the bacteria from early stationary phase cultures into ddH_2_O, and then quantified the numbers of viable bacteria every 30 min over the course of 2 h. Titers of UTI89*ΔmiaA* carrying the empty vector pACYC184 were greatly reduced following exposure to hypoosmotic stress, whereas the levels of UTI89/pACYC184 and UTI89Δ*miaB*/pACYC184 remained mostly unchanged (Figure 3A). Survival of UTI89Δ*miaA* was restored by complementation with pMiaA_nat_. To ensure that reduced survival of UTI89Δ*miaA* was attributable to hypoosmotic stress and not starvation, cells were resuspended in ddH_2_O containing 0.1% glucose, which is comparable to the glucose levels within our M9 medium. Viable bacteria measured after 120 min indicated that the death of UTI89Δ*miaA* was not due to nutrient deprivation (Figure 3B). We also observed that UTI89Δ*miaA* grew poorly in hyperosmotic conditions, created by addition of 5% NaCl to standard LB, whereas UTI89Δ*miaB* behaved more like the wild-type strain (Figure 3C). Growth of UTI89Δ*miaA* was restored to wild-type levels by complementation with pMiaA_nat_ (Figure 3D). These results indicate that the *miaA* knockout has decreased resistance to both hypo- and hyperosmotic stresses.

### Hyperosmotic stress attenuates MiaA translation

**Figure 4.**
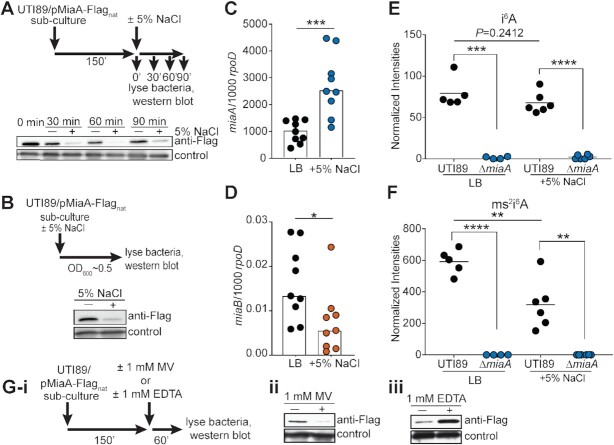
High salt stress downregulates MiaA translation and reduces ms^2^i^6^A levels. (**A**) Top panel shows schematic of the experimental setup. UTI89/pMiaA-Flag_nat_ was diluted from overnight cultures into fresh LB and grown shaking for 2.5 h at 37°C prior to resuspension in LB or LB + 5% NaCl. Incubations were continued for the indicated times before samples were collected and analyzed by western blots using anti-Flag and anti-*E. coli* (loading control) antibodies (bottom panel). (**B**) Top panel summarizes the experimental setup in which overnight cultures of UTI89/pMiaA-Flag_nat_ were diluted directly into fresh LB or LB + 5% NaCl and grown shaking to OD_600_ of 0.5 prior to processing for western blot analysis (bottom panel). (**C** and **D**) UTI89 from mid-logarithmic cultures in LB was resuspended in LB or LB + 5% NaCl and 1 h later the levels of *miaA* and *miaB* transcripts were determined by RT-qPCR. Bars indicate mean values from 9 independent replicates, each with two technical replicates. *P< 0.05; ***P< 0.001 by Mann–Whitney *U* tests. (**E** and **F**) Graphs show normalized levels of i^6^A and ms^2^i^6^A recovered from UTI89 and UTI89Δ*miaA* following growth to OD_600_ of 0.5 in LB or LB + 5% NaCl, as determined by LC–MS. **P< 0.01; ***P< 0.001; ****P < 0.0001 by unpaired *t* tests. Bars indicate median values from four to six independent replicates. (**G(i)**) Schematic of the experimental setup in which 1 mM (**ii**) MV or (**iii**) EDTA were added to UTI89/pMiaA-Flag_nat_ cultures for 1 h prior to processing for western blot analyses.

We next examined how MiaA levels in UTI89 change in response to environmental cues, focusing on hyperosmotic stress. For these assays, we employed a low-copy number plasmid (pMiaA-Flag_nat_) that encodes C-terminal FLAG-tagged MiaA under control of the native *miaA* promoter. Mid-logarithmic phase cultures of UTI89/pMiaA-Flag_nat_ were resuspended in LB ± 5% NaCl and levels of MiaA-Flag were then assessed by western blot at 30-min intervals over the course of 1.5 h (Figure 4A). Interestingly, MiaA levels in UTI89 exposed to high salt broth were decreased at all time points in comparison with bacteria grown in standard LB. We observed a similar phenomenon if overnight cultures of UTI89/pMiaA-Flag_nat_ in standard LB were back-diluted into high salt broth and then grown to mid-logarithmic phase (OD_600_ ≈ 0.5, Figure 4B). Importantly, in these assays we observed no loss of the pMiaA-Flag_nat_ construct. In addition, *miaA* transcripts were often elevated following exposure of UTI89 to high salt (Figure 4C), suggesting that the downregulation of MiaA protein levels in response to this osmotic stress occurs via a post-transcriptional mechanism. The transcription of *miaB* was generally reduced under the same conditions (Figure 4D).

To determine if lower amounts of the MiaA protein detected in high salt broth culture affected i^6^A or ms^2^i^6^A levels, we employed liquid chromatography–coupled mass spectrometry (LC–MS). Normalized amounts of the i^6^A modification in wild-type UT89 grown to mid-logarithmic phase in LB were similar to those measured in UTI89 grown in high salt broth (Figure 4E). However, hyperosmotic stress caused a marked reduction in ms^2^i^6^A levels (Figure 4F), possibly due to reduced transcription of *miaB* (Figure 4D). i^6^A and ms^2^i^6^A were undetectable in UTI89Δ*miaA*, regardless of high salt exposure, confirming that MiaA is required for both modifications (Figure 4E and F). In contrast, deletion of *miaB* prevented formation of ms^2^i^6^A, but led to greatly elevated levels of i^6^A (Supplementary Figure S6). Cumulatively, these data indicate that in response to hyperosmotic stress UTI89 can post-transcriptionally downregulate MiaA, coordinate with reduction of both *miaB* messages and ms^2^i^6^A levels. We observed that MiaA levels were also reduced upon exposure to MV (Figure 4G-i, ii), indicating that MiaA levels can be varied in response to distinct stressors. Interestingly, MiaA levels were intensified in the presence of the divalent metal chelator EDTA (Figure 4G-iii), raising the possibility that the quantities of this tRNA modifying enzyme are controlled by one or more EDTA-sensitive metalloproteases as previously suggested for the tRNA modifier GidA (81). This idea requires further testing, as the chelating activity of EDTA can potentially impact multiple cellular processes that require divalent metals.

### Overexpression of MiaA is detrimental under stressful conditions

**Figure 5.**
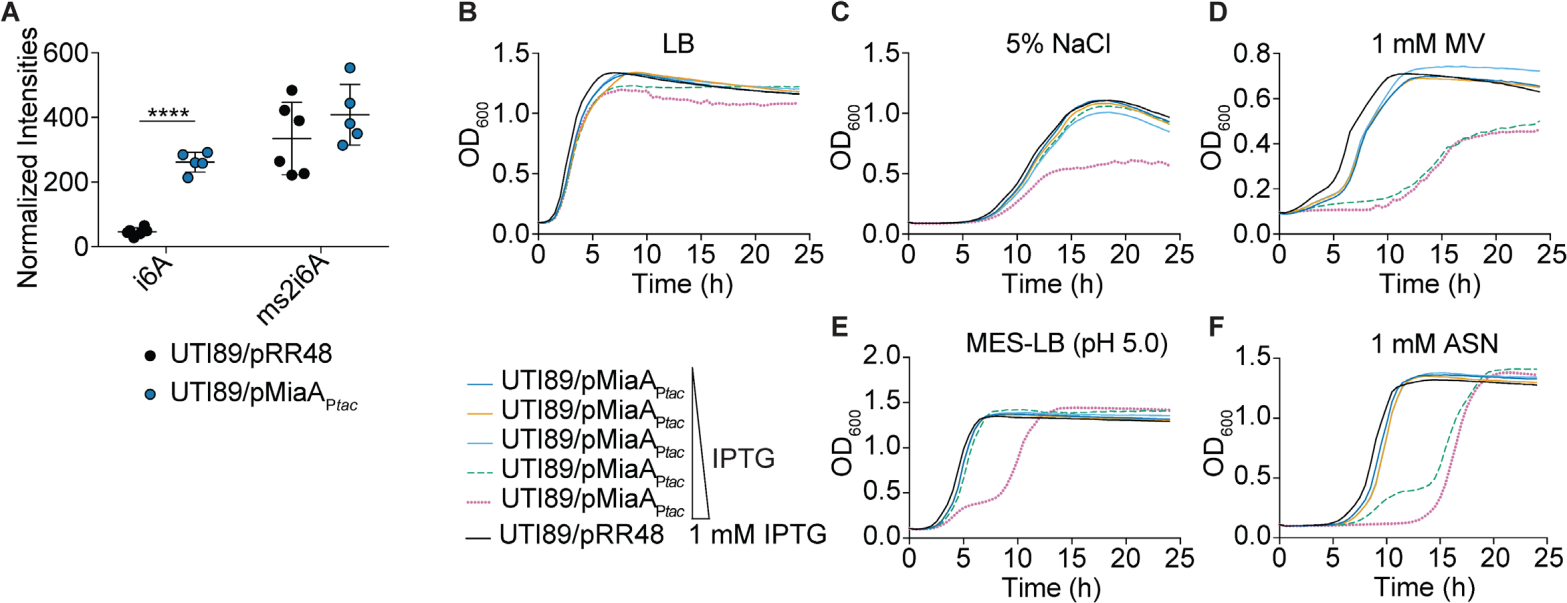
Overexpression of MiaA reduces ExPEC stress resistance. (**A**) Relative levels of i^6^A and ms^2^i^6^A in UTI89 carrying either pMiaA_P_*_tac_* or the empty vector control pRR48 following growth to OD_600_ of 0.5 in LB with 1 mM IPTG, as quantified by LC-MS. ****P < 0.0001 by unpaired *t* test; *n* = 5 to 6 independent replicates per group. (**B–F**) Curves depict growth of UTI89 carrying pMiaA_P_*_tac_* or the empty vector control pRR48 in LB, LB + 5% NaCl, LB + 1mM MV, MES-LB, or MES-LB + 1 mM ASN. Cultures were grown shaking at 37°C with IPTG added in 10-fold increments from 0 to 1000 μM, as indicated. Curves depict mean values from four replicates and are representative of three independent experiments.

Since it was unexpected that high salt stress would lead to a decrease in the levels of MiaA and the ms^2^i^6^A modification, we set out to determine if overexpression of MiaA would affect bacterial growth during environmental stress. To overexpress MiaA, we utilized a plasmid (pRR48) with *miaA* under control of an IPTG-inducible P*tac* promoter in wild-type UTI89. By LC-MS, normalized intensities of i^6^A were significantly higher in UTI89/pMiaA_P_*_tac_* induced with 1 mM IPTG and grown to mid-logarithmic phase in LB compared to UTI89 carrying the empty vector pRR48, whereas the amounts of ms^2^i^6^A were only modestly elevated (Figure 5A).

Next, overnight cultures of UTI89/pMiaA_P_*_tac_* were back-diluted into LB, LB + 1 mM MV, LB + 5% NaCl, MES-LB, or MES-LB + 1 mM ASN, and grown in the presence of increasing IPTG concentrations (Figure 5B–F). Lower levels of MiaA protein induction caused no overt defects and the bacteria grew much like UTI89/pRR48. However, higher levels of IPTG-induced MiaA expression hindered growth of UTI89/pMiaA_P_*_tac_* in the presence of 1 mM MV, 5% NaCl, MES-LB, and 1 mM ASN. In contrast, over expression of MiaB did not affect bacteria growth in these *in vitro* assays (Supplementary Figure S7). These findings indicate that too much MiaA can be detrimental to bacterial fitness, similar to the complete absence of the enzyme.

### Both deletion and overexpression of MiaA increase frameshifting

**Figure 6.**
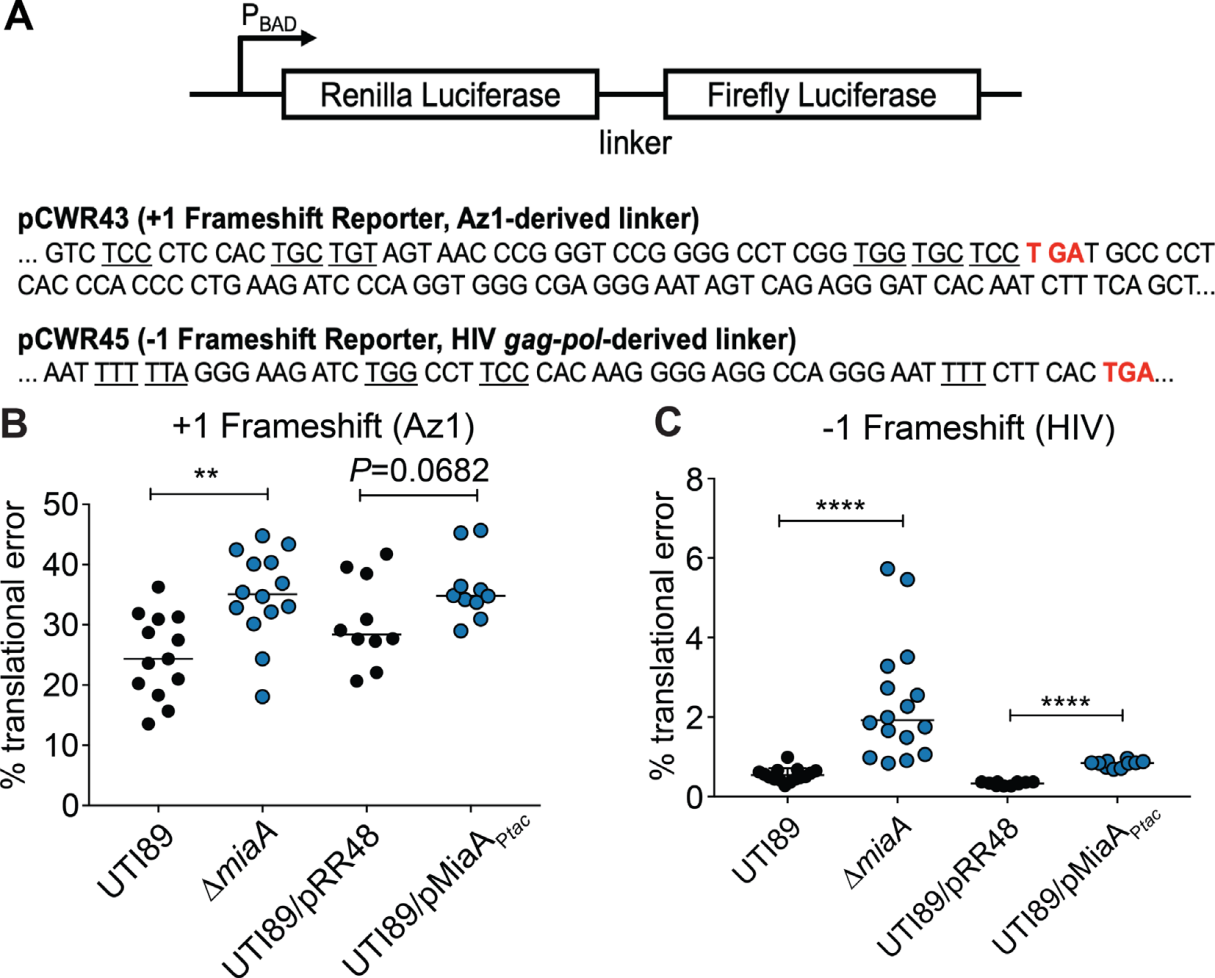
Changing levels of MiaA increase frameshifting. (**A**) Diagram depicts the structures of the dual luciferase reporters, with specific intergenic linker sequences and premature stop codons (red) indicated below. Each linker contains multiple MiaA-sensitive UNN codons (underlined). (**B** and **C**) Graphs show results from + 1 and –1 frameshifting assays with UTI89, UTI89Δ*miaA*, UTI89/pRR48, or UTI89/pMiaA_P_*_tac_* carrying one of the dual luciferase reporter constructs. Bacteria were grown shaking at 37°C in LB, with 1 mM IPTG included for UTI89/pRR48 and UTI89/pMiaA_P_*_tac_*. After reaching an OD_600_ of ∼0.2, 0.2% arabinose was added to induce expression of the luciferases. At an OD_600_ of 0.5, translational error rates were quantified by determining the ratio of firefly to renilla luciferase activities in bacteria carrying the +1 (Az1) and –1 (HIV) reporter constructs. Results were normalized using the ratio of firefly to renilla luciferase activity in bacteria carrying control plasmids in which the luciferases are in-frame. **P< 0.01; ****P < 0.0001 by two-tailed unpaired *t* tests; *n* = 10–14 independent replicates.

Previous research in K-12 *E. coli* and *Salmonella* showed that deletion of *miaA* can compromise translational fidelity, resulting in increased ribosomal frameshifting (82,83). To determine the effects of MiaA on frameshifting in UTI89, we utilized dual-luciferase reporter plasmids that consist of a translational fusion of firefly luciferase downstream of renilla luciferase (Supplementary Table S1). Linker sequences, derived from either Antizyme 1 (Az1) or HIV *gag-pol*, were placed between the two luciferase genes (Figure 6A). The Az1-derived linker sequence contains a stop codon positioned in-frame so that a + 1 frameshift must occur for read-through expression of firefly luciferase (41). In contrast, a –1 frameshift is required for expression of firefly luciferase downstream of the HIV-derived linker (40). Importantly, upstream of the in-frame stop codons in both linkers are UNN codons that can be recognized by MiaA-modified tRNAs. The firefly and renilla luciferases act on distinct substrates, which are used to sequentially assess levels of expression of each enzyme (40,41). Control plasmids in which the two luciferases are in-frame were used to normalize the data by accounting for ribosome drop-off.

To examine the consequences of MiaA expression on frameshifting, the dual-luciferase reporter constructs were used in combination with wild-type UTI89, UTI89Δ*miaA*, UTI89/pMiaA_P_*_tac_*, and UTI89 carrying the empty control vector pRR48. After overnight growth in LB, UTI89 and UTI89Δ*miaA* were back-diluted into LB while UTI89/pMiaA_P_*_tac_* and UTI89/pRR48 were back-diluted into LB + 1 mM IPTG to induce MiaA expression. After reaching mid-log growth, the enzymatic activities of the two luciferases were quantified. Both the lack of MiaA and MiaA overexpression caused notable increases in frameshifting in both the +1 and –1 directions (Figure 6B and C). These results confirm that loss of MiaA can increase frameshifting and show that elevated MiaA levels can likewise impact the fidelity of translation.

### Changing levels of MiaA alters the spectrum of expressed proteins

**Figure 7.**
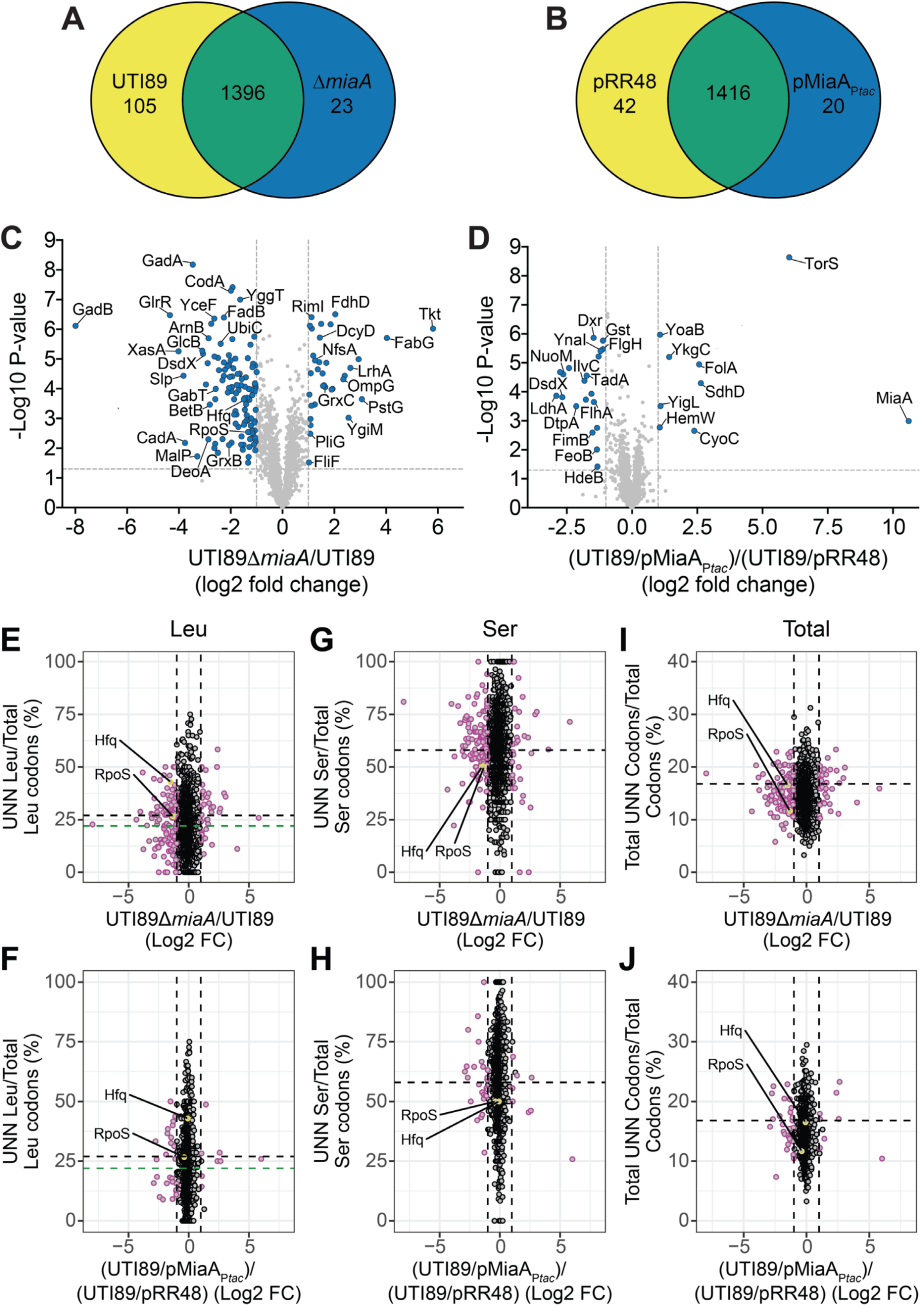
Altering MiaA levels changes the spectrum of expressed proteins. (**A** and **B**) Venn diagrams indicate numbers of unique and shared proteins detected in wild-type UTI89 versus UTI89Δ*miaA*, or in UTI89/pRR48 versus UTI89/pMiaA_P_*_tac_*, following growth to OD_600_ of 0.5 in LB. IPTG (1 mM) was included in the UTI89/pRR48 and UTI89/pMiaA_P_*_tac_* cultures. Relative protein levels were determined by MudPIT. (**C** and **D**) Volcano plots show relative protein levels (Log2-fold change) versus *P* values (–Log10). Vertical dashed lines denote 2-fold change cutoffs, while the horizontal dashed lines indicate a *P* value of 0.05. Blue dots, proteins that were significantly changed (*P*< 0.05) by at least 2-fold. *P* values were determined by Student's *t* tests; *n* = 4 independent replicates for each group. (**E–J**) Plots show relative protein levels (Log2-fold change as noted in C and D) versus UNN codon usage ratios for Leu, Ser, or total UNN codons per open reading frame. For I and J, total codons refer specifically to those for Leu, Ser, Cys, Phe, Trp and Tyr. Purple circles, proteins that were significantly changed (*P*< 0.05, by Student's *t* tests) by at least 2-fold in UTI89Δ*miaA* or UTI89/pMiaA_P_*_tac_* relative to their controls. Positions for Hfq and RpoS are highlighted (yellow). The vertical dotted lines are placed at the 2-fold change cutoffs. Black and green horizontal dashed lines specify the mean UNN codon usage ratios for all open reading frames encoded by UTI89 and the K-12 strain MG1655, respectively.

To determine how deletion and overexpression of MiaA affect translation we used multidimensional protein identification technology (MudPIT; LCMS/MS) with wild-type UTI89 and UTI89Δ*miaA* cultures grown to mid-log phase in LB, and UTI89/pMiaA_P_*_tac_* and UTI89/pRR48 similarly grown in LB + 1 mM IPTG. Of 1524 proteins detected in UTI89 and UTI89Δ*miaA*, 105 were picked up only in the wild-type strain and 23 were unique to the *miaA* knockout mutant (Figure 7A). 1,471 proteins were identified in UTI89/pRR48 and UTI89/pMiaA_P_*_tac_*, with 42 being exclusive to UTI89/pRR48 and 20 seen only in the MiaA overexpression strain (Figure 7B). 115 proteins were significantly downregulated in UTI89Δ*miaA* relative to wild-type UTI89, while 34 proteins were upregulated in the knockout mutant (Figure 7C). Notably fewer proteins were significantly altered when MiaA was overexpressed. Relative to the control strain UTI89/pRR48, 20 proteins were downregulated in UTI89/pMiaA_P_*_tac_*, whereas nine (including MiaA) were upregulated (Figure 7D).

The specific proteins detected, including those that were differentially expressed due to *miaA* deletion or overexpression, are detailed in Supplementary Dataset S1. The differentially expressed proteins were assigned to one or more of 14 functional categories (see *Categories* worksheet and embedded graph in Supplementary Dataset S1). A majority of the altered proteins were linked with metabolic pathways, secondary metabolites, and functions associated with the bacterial envelope. These included several proteins involved in sugar and fatty acid metabolism and the biosynthesis and regulation of electron transport chains (e.g. UbiC, WrbA, ChrR, Qor, NuoM, NudJ and CyoC). The dysregulation of these factors likely contributed to the various phenotypic defects observed in our *in vitro* and *in vivo* assays and suggested MiaA involvement in other important processes.

In particular, many of the differentially expressed proteins were shown in previous studies to directly or indirectly affect motility or biofilm development. The former group comprised the chemotaxis protein CheA and the flagella-associated proteins FliF, FlhA and FlgH. Accordingly, both deletion of the *miaA* gene and MiaA overexpression markedly decreased UTI89 motility on swim plates (Supplementary Figure S8). MiaB did not affect motility in these assays. Factors linked with biofilm development include YoaB, the type 1 pilus-associated regulator FimB and periplasmic chaperone FimC, the acid stress-response chaperone HdeB, the cellulose synthase catalytic subunit BcsA, and the cytochrome *bo* subunit CyoC. Using yeast extract-casamino acids (YESCA) medium, which promotes the development of elaborate rugose-colony biofilms (47,84), we found that UTI89Δ*miaA*, but not UTI89Δ*miaB*, formed atypical biofilms with notably less rugosity than the parent strain (Supplementary Figure S9). Interestingly, the biofilms formed by UTI89Δ*miaA* were architecturally similar to those formed by a UTI89 mutant lacking the CyoC-interacting partners CyoAB (47).

Our MudPIT results also indicated that MiaA can regulate numerous proteins that have been associated with redox and bacterial responses to nitrosative, oxidative, and more generally, genotoxic stresses (Supplementary Dataset S1). Aberrant expression of these factors, including proteins like GadB, CadA, Dps, glutathione S-transferase Gst, and the glutatredoxins GrxB and GrxC, may account for increased sensitivity to oxygen and nitrogen radicals (see Figure 2D–H and Figure 5D–F). Some of these factors, and others like HdeA and HdeB, can also guard against acid stress. Follow-up experiments confirmed that UTI89Δ*miaA*, but not UTI89Δ*miaB*, is notably less resistant to acid stress than the wild-type strain (Supplementary Figure S10). On average, relative to wild-type UTI89, UTI89Δ*miaA* titers were reduced over 6,000-fold following exposure to acidic conditions in LB.

The sensitivity of both UTI89Δ*miaA* and UTI89/pMiaA_P_*_tac_* to osmotic stress may arise due to the significant downregulation of proteins like SLP, BetB, YggT, ProP, and YnaI (Supplementary Dataset S1). Other differentially expressed proteins that probably contribute to the varied phenotypes associated with *miaA* deletion or MiaA overexpression in our assays include multiple transcriptional regulators, several ribosome- and RNA-associated factors, and the tRNA ligases LysU, TyrS, and PheS. These findings indicate that MiaA is tied into a complex web of factors that can have direct and indirect effects on translation. Driving this point home is the observation that MiaA overexpression suppresses the production of TadA, an enzyme that catalyzes the deamination of adenosine-to-inosine (A-to-I) in Arg2 tRNA and a select set of mRNAs (85,86). In K-12 *E. coli*, the A-to-I editing function of TadA can recode at least 12 mRNAs, which results in the generation of proteins with altered activities that can impact bacterial cell physiology (86). Among the known TadA-edited transcripts is one encoding IlvC, an enzyme involved in isoleucine and valine biosynthesis which, like TadA, is downregulated ∼3.5-fold in UTI89 when MiaA is overexpressed (Figure 7D).

### UTI89Δ*miaA* phenotypes are not entirely due to aberrant RpoS or Hfq expression

In K-12 *E. coli*, the deletion of *miaA* results in decreased translation of the alternate Sigma factor RpoS (σ^S^) and the small RNA chaperone Hfq (7,22,23). Both of these factors are important for the stress resistance and virulence potential of ExPEC (68,87). In line with results from K-12 *E. coli*, our proteomics analysis indicated that RpoS and Hfq levels were reduced 2.5- and 2.8-fold, respectively, in UTI89Δ*miaA* relative to the wild-type strain (Figure 7C and Supplementary Dataset S1). RpoS downregulation in the absence of *miaA* was also confirmed by western blot analysis (Supplementary Figure S11A) These observations suggest that the phenotypic defects associated with UTI89Δ*miaA* might be attributable to aberrant expression of RpoS or Hfq. However, despite some similarities, the phenotypes that we previously observed with UTI89 mutants lacking either *rpoS* or *hfq* are distinct from one another and from those that we report here with UTI89Δ*miaA* (68,87). Furthermore, the induced expression of recombinant RpoS or Hfq failed to rescue growth of UTI89Δ*miaA* under hyperosmotic conditions (Supplementary Figure S11B-E). The pRpoS_P_*_tac_* and pHfq_P_*_tac_* expression constructs used in these assays can complement UTI89 mutants lacking *rpoS* or *hfq*, respectively (68,87). Cumulatively, these data indicate that the phenotypes seen with UTI89Δ*miaA* are not entirely due to attenuated expression of either RpoS or Hfq. Also of note, our ability to complement UTI89Δ*miaA* with MiaA expression constructs (see Figures 2 and 3, and Supplementary Figure S9) demonstrates that the phenotypic defects associated with this knockout mutant are not caused by off target mutations or polar effects on *hfq*, which lies immediately downstream of *miaA*.

### UNN codon usage by MiaA-sensitive transcripts

Messages like those encoded by *rpoS* and *hfq* are classified as Modification Tunable Transcripts (MoTTs), which are identifiable by 1) codon usage different from that of average transcripts and 2) translation that is sensitive to changing levels of tRNA modifications (13,23). Studies in the K-12 *E. coli* strain MG1655 of *rpoS*, *hfq*, and other transcripts suggest that MiaA-sensitive MoTTs have higher than average ratios of UNN-Leu codons relative to total Leu codons (22,23). This led us to ask if UNN-Leu codon usage correlates with protein expression levels in UTI89 when MiaA is either absent or over-produced. Plotting results from our MudPIT analysis versus UNN-Leu codon usage (Supplementary Dataset S1) showed that just over 60% of the proteins that are differentially expressed in UTI89Δ*miaA* or UTI89/pMiaA_P_*_tac_* have UNN-Leu codon usage ratios that are greater than the K-12 average ratio of 0.22 (Figure 7E-F, green dashed line). In agreement with previous findings (22,23), RpoS and Hfq are among the differentially expressed proteins with UNN-Leu codon usage ratios of greater than 0.22. However, the average UNN-Leu codon usage ratio in UTI89 is somewhat higher than that in K-12 *E. coli*. Using this value, which is 0.28, less than half of the proteins that are differentially regulated in UTI89Δ*miaA* or UTI89/pMiaA_P_*_tac_* have greater than average UNN-Leu codon usage ratios (Figure 7E-F, black dashed line). Furthermore, among the proteins that are not significantly altered by either deletion or overexpression of *miaA*, about 30% have UNN-Leu codon usage ratios greater than 0.28. By calculating Spearman's rank correlation coefficients (ρ), we found a significant, though modest negative correlation between protein abundance and UNN-Leu codon usage ratios for UTI89Δ*miaA* (ρ = –0.07, *P* = 0.014), but not for the MiaA overexpression strain UTI89/pMiaA_P_*_tac_* (ρ = –0.05, *P* = 0.153). Thus, higher UNN-Leu codon usage ratios within an open reading frame correlate with reduced protein expression in the absence of MiaA. Additional analyses of the MiaA-sensitive codons for Ser, Cys, Phe, Trp and Tyr (Figure 7G and H, Supplementary Figure S12), or of all UNN codons (Figure 7I and J), revealed no clear trends linking other UNN codon usage ratios and protein abundance in either UTI89Δ*miaA* or UTI89/pMiaA_P_*_tac_*.

Building on these observations, we next assessed if changes in protein abundance due to MiaA deletion or overexpression were affected by the numbers of single or consecutive UNN codons within each open reading frame, but not UNN codon usage ratios per se (see Supplementary Dataset S1). Spearman's rank correlation coefficients indicated significant negative correlations between protein abundance in UTI89Δ*miaA* and the presence of one, two, or three consecutive UNN codons (Supplementary Table S3). Similar trends were detected in UTI89/pMiaA_P_*_tac_*. The correlations were slight when considering the less frequent instances in which four or more consecutive UNN codons were present. Negative correlations between protein abundance and total UNN codons per open reading frame were also evident in UTI89Δ*miaA* and especially UTI89/pMiaA_P_*_tac_*, independent of UNN positions within the coding sequences. Cumulatively, these results indicate that higher numbers of UNN codons, especially singlet and doublet UNN codons, may render translation of specific transcripts more sensitive to varying MiaA levels.

### Conditional negative effects of DMAPP binding when MiaA levels are elevated

**Figure 8.**
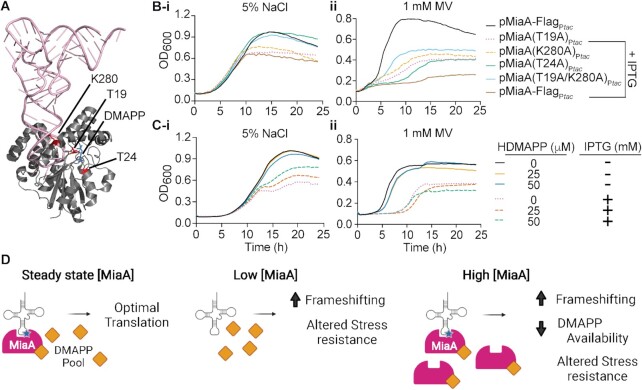
Conditional adverse effects of increased DMAPP binding by MiaA. (**A**) Crystal structure-based model of MiaA (grey) complexed with tRNA (pink) and DMAPP (blue). The three residues that were mutated to alanines are indicated. Image created using PyMOL with structural information (2ZXU) downloaded from the RCSB Protein Data Bank (125). (**B**) Graphs show growth curves for recombinant UTI89 strains with the specified plasmids in (**i**) LB with 5% NaCl (left) or (**ii**) 1 mM MV (right) ±1 mM IPTG, as indicated. (**C**) Growth curves for UTI89/pMiaA_P_*_tac_* in (**i**) LB with 5% NaCl (left) or (**ii**) 1 mM MV (right) ± HDMAPP and IPTG, as noted. Each curve shows average values from four replicates and are representative of three independent experiments. (**D**) A working model of how changing levels of MiaA can impact translational fidelity and stress resistance. Created with Biorender.com.

To better understand why overexpression of MiaA, but not MiaB, rendered UTI89 more sensitive to stress (see Figure 5 and Supplementary Figure S7), we introduced a series of point mutations into MiaA that are known to alter the substrate binding and catalytic activities of the enzyme (88). A crystal structure-based model of MiaA in complex with tRNA and the prenyl group precursor DMAPP is shown in Figure 8A, with the mutated residues indicated. The T19A mutation is expected to decrease the catalytic activity (*K*_cat_) of MiaA by ∼600-fold relative to the wild-type enzyme, with little effect on the Michaelis constants (*K*_m_) for either tRNA (*K*_m_*^RNA^*) or DMAPP (*K*_m_*^DMAPP^*) (88). In contrast, the T24A mutation increases the *K*_m_*^RNA^* 10–20 times higher than wild-type MiaA and elevates *K*_m_*^DMAPP^* by more than 200-fold. The K280A mutation causes >30-fold increases in both *K*_m_^RNA^ and *K*_m_^DMAPP^ values. We also generated a double T19A/K280A mutant that is expected to bind tRNA and DMAPP poorly and completely lack catalytic activity. Mutations were introduced into plasmid pMiaA-Flag_P_*_tac_*, encoding IPTG-inducible MiaA with a C-terminal Flag tag. The wild-type and mutant MiaA variants were all expressed at similar levels in UTI89 following IPTG induction (Supplementary Figure S13).

As expected from results presented in Figure 5, IPTG-induced overexpression of Flag-tagged wild-type MiaA markedly attenuated growth of UTI89 in the presence of both high salt and MV (Figure 8B). In high salt, overexpression of the K280A and catalytically inactive T19A MiaA mutants were nearly as detrimental to bacterial growth as wild-type MiaA. In contrast, overexpression of the T24A and T19A/K280A mutants had no adverse effects on UTI89 growth in high salt. In MV, the overexpression of all four MiaA mutants impaired growth of UTI89, but none were as detrimental as overexpression of wild-type MiaA. These results suggest that high-level expression of MiaA can impair bacterial fitness via distinct mechanisms, depending on the specific environmental stressors present.

Data presented in Figure 8B argue that the attenuated growth of UTI89 caused by MiaA overexpression in high salt is mostly attributable to increased binding of DMAPP. In addition to serving as a precursor for MiaA-mediated tRNA modifications, DMAPP is also an important cellular metabolite for a variety of other biosynthetic pathways (89,90). To assess the possibility that reduction of DMAPP precursor pools contributed to some of the observed defects caused by MiaA overexpression, we performed growth curve analyses of UTI89/pMiaA_P_*_tac_* using media supplemented with DMAPP or its precursor HDMAPP. The addition of DMAPP, which is highly membrane impermeable (91), had no discernable effects. However, in high salt media with IPTG present to induce MiaA overexpression, addition of the DMAPP precursor HDMAPP rescued growth of UTI89/pMiaA_P_*_tac_* in a dose-dependent manner (Figure 8C). Added HDMAPP did not have an effect on growth of UTI89/pMiaA_P_*_tac_* in the absence of IPTG, and failed to rescue bacterial growth when MiaA was overexpressed in the presence of MV. These results are in line with observations made with the MiaA point mutants (Figure 8B), suggesting that the UTI89 growth defects associated with MiaA overexpression in high salt media, but not with MV, are in large part caused by depletion of DMAPP pools.

## DISCUSSION

The results presented here demonstrate that MiaA is crucial for ExPEC fitness and virulence, and that changing MiaA levels can impact the translation of a broad spectrum of proteins. Our findings are in agreement with previously published work showing that tRNA modifying enzymes can influence the fitness and virulence potential of a variety of microbial pathogens (6,11,92–104). The attenuation of bacterial virulence-related phenotypes in the absence of a specific tRNA modifying enzyme can, in some cases, be explained by sub-optimal translation of specific toxins or key regulatory factors (11,102–104). For example, deletion of *miaA* in the diarrheagenic bacteria *Shigella flexneri* ablates translation of the transcriptional master regulator VirF, resulting in the reduced expression of downstream virulence factors (95,105). Overexpression of recombinant VirF alone is sufficient to rescue the *miaA* mutant, suggesting that low-level production of VirF is in large part responsible for the virulence-related defects caused by the deletion of *miaA* in *S. flexneri*. In contrast, our work indicates that the diverse phenotypes affected by MiaA expression in the ExPEC isolate UTI89 are not attributable to any single factor, but rather arise due to the altered expression and dysregulation of multiple proteins and pathways downstream of MiaA.

The ms^2^i^6^A modification is understood to affect the fidelity of translation (5,8,19). Earlier work in lab-adapted K-12 *E. coli* and *Salmonella* strains showed that bacteria lacking *miaA* have an increase in the + 1 direction of frameshifting, but not in the –1 direction (82,83). In these studies, the i^6^A modification was found to be a major contributor to ribosome fidelity. In UTI89, significant increases in frameshifting were seen in both the + 1 and –1 directions when *miaA* was knocked out. When MiaA was overproduced, we also observed marked elevation of frameshifting in the –1 direction, while frameshifting levels in the +1 direction were more modest. Most reports to date indicate that tRNA modifications typically affect frameshifting primarily in one direction (5,83). At first glance, our data seemingly counter this trend. However, we note that the expression of firefly luciferase downstream of the HIV-derived linker in our reporter system may also occur as a consequence of a +2 frameshift, rather than a –1 frameshift, which would more closely mirror what was observed in previous studies with K-12 *E. coli* and *Salmonella* strains (82,83). It is also possible that the apparent increases in both –1 and +1 frameshifting observed in our assays reflect the presence of MiaA-sensitive regulatory circuits in UTI89 that are different from those in lab-adapted K-12 *E. coli* or *Salmonella* strains.

During the course of this study, we were surprised to observe that MiaA levels in the ExPEC reference strain UTI89 were substantially decreased in response to high salt and other stressors like MV (see Figure 4). Due to the sweeping phenotypes seen in the absence of MiaA, we hypothesized that the levels of MiaA would have stayed the same or increased in response to stressors like high salt and MV. However, our data indicate that MiaA levels are fine-tuned within ExPEC such that too much or too little enzyme can have similarly detrimental consequences. The effects of MiaA expression in our growth curve assays were dose–dependent, with high-level expression of MiaA being nearly as disruptive as the deletion of *miaA*. For instance, low-level expression of MiaA restored the resistance of UTI89Δ*miaA* to high salt, whereas overexpression of MiaA resulted in greatly increased sensitivity (see Figure 5). Defective growth of UTI89 caused by overexpression of MiaA during high salt stress was offset if the enzyme was mutated to disrupt DMAPP binding or if the media was supplemented with the DMAPP precursor HDMAPP (see Figure 8A–C). These observations suggest that the growth defects associated with MiaA overexpression in high salt media are largely attributable to depletion or sequestration of DMAPP. This molecule feeds into a number of other critical pathways, including the biosynthesis of quinones within the electron transport system (90). Interestingly, the growth defects caused by MiaA overexpression in the presence of MV were not linked with DMAPP availability and were only partially countered if the DMAPP binding and catalytic activities of MiaA were ablated. In these assays, the overexpression of DMAPP-binding and catalytically inactive MiaA mutants may still impair ExPEC growth in the presence of MV due to the ability of MiaA to form multimers (28). It is feasible that the formation of mixed wild-type and mutant MiaA complexes could in effect take the wild-type enzyme out of action, partially mimicking the phenotype of a *miaA* deletion mutant. Overall, these observations indicate that MiaA overexpression can interfere with bacterial growth via distinct mechanisms, dependent upon the types of environmental stressors that are present.

MiaA is part of a complex superoperon and is regulated directly and indirectly by a variety of factors, including RNaseE, Hfq, RpoS and the heat shock sigma factor RpoH (106–108). However, the control of MiaA expression, and the regulation of tRNA modifying enzymes in general, is not clearly understood (10,109). Though we did not investigate MiaA regulation in detail here, our RT-qPCR experiments indicate that MiaA levels are reduced in response to high salt stress via a post-transcriptional mechanism (Figure 4A-D). Interestingly, *miaA* has a higher-than-average UNN Leu codon usage ratio of 0.46, suggesting that MiaA may help regulate the translation of its own transcripts (23). Furthermore, we note that MiaA levels are also reduced in response to oxidative stress caused by MV, but intensified in the presence of the metal chelator EDTA (Figure 4G). The latter observation suggests that MiaA quantities can be controlled by one or more EDTA-sensitive metalloprotease, as proposed for the tRNA modifier GidA (81). Additional work is needed to decipher how proteolytic degradation and other post-transcriptional processes might modulate MiaA levels in response to specific stressors like MV and high salt.

By adjusting the levels of tRNA modifying enzymes like MiaA, ExPEC and other organisms may be able to vary the diversity of translated proteins and thereby optimize adaptive responses to stressful stimuli (9–12,81,110–112). Indeed, the overexpression and deletion of *miaA* led to the generation of distinct proteomes by UTI89 (Figure 7) and compromised the ability of this ExPEC strain to deal with multiple stressors (Figure 2, 3 and 5). Because tRNA modifications can have pleiotropic effects, it is not always easy to distinguish the direct and indirect effects that tRNA modifying enzymes like MiaA have on translation (12). For example, pioneering work in K-12 *E. coli* indicates that the efficient translation of RpoS and Hfq relies on MiaA for proper decoding of UNN-Leu codons (7,22,23), but these factors can themselves regulate the expression of numerous other proteins, including MiaA itself (106,113–115). The capacity for MiaA to have additional, indirect effects on the fidelity and specificity of translation is further highlighted by our proteomics data showing that MiaA impacts the expression of multiple ribosome- and RNA-associated factors, tRNA ligases, and the RNA editing enzyme YfhC (TadA). These findings suggest the existence of a complex network of RNA and translational modifiers that can regulate the expression of one another. Layered on top of this is the potential for MiaA to affect the biosynthesis and availability of specific metabolites used by other tRNA modifying enzymes (10,98).

Increases in frameshifting due to changing levels of MiaA may also allow for more error-prone translation and the subsequent diversification of expressed proteins, which could allow bacteria to better deal with stressful stimuli. The ability of cells to actively regulate frameshifting and other translational errors in order to generate mutant proteins that deviate from those encoded by the genome is gaining appreciation as an adaptive response to stress (116–119). Ongoing studies aim to utilize ribosomal profiling along with RNA-seq and proteomics to determine if off-frame and mutant proteins are being produced by ExPEC via translational modifiers like MiaA in response to stressful stimuli. Similar lines of research may also shed light on the somewhat more cryptic functions of the MiaB-catalyzed tRNA modification.

In the absence of *miaA*, the i^6^A and ms^2^i^6^A modifications are not detectable (Figure 4E and F), as expected from previously published work (109). When MiaA is overproduced, high levels of i^6^A are observed, while ms^2^i^6^A modification levels remain relatively stable (Figure 5A). Interestingly, high salt stress reduces both MiaA expression and ms^2^i^6^A levels but does not significantly affect i^6^A levels (Figure 4E and F). In contrast, the disruption of *miaB* prevents the formation of ms^2^i^6^A and causes marked increases in i^6^A levels (see Supplementary Figure S6), but this had no phenotypic effect in any of our assays. These findings present a conundrum—why does high-level production of i^6^A due to MiaA overexpression attenuate the stress resistance of UTI89 while even higher levels of i^6^A that build up in the absence of MiaB had no overt phenotypic effects in our assays? In considering this issue, it should be noted that we quantified normalized amounts of i^6^A and ms^2^i^6^A, and not specific tRNAs, leaving open the possibility that changing levels of MiaA differentially affect distinct subsets of cognate or potentially non-cognate tRNAs. Either possibility could help account for the contrasting phenotypic effects linked with elevated i^6^A levels due to MiaA overexpression versus those caused by *miaB* deletion. However, our data indicate that during high salt stress, the growth defects associated with MiaA overexpression are mostly due to disruption of DMAPP pools, as noted above. The deletion of *miaB*, by causing a buildup of i^6^A rather than accelerated production of this modified residue, may have less of an abrupt impact on the availability of substrates like DMAPP. Finally, it is feasible that MiaA has moonlighting function(s), affecting non-tRNA targets and compromising bacterial fitness when produced in excess.

Cumulatively, the findings presented here, and summarized in Figure 8D, highlight the central and complex roles that core metabolic genes like *miaA* can have on the elaboration and fine-tuning of pathogen stress resistance and virulence-associated phenotypes. By varying levels of MiaA, our results show that ExPEC can markedly alter frameshifting frequencies and the spectrum of expressed proteins, directed in part by the prevalence of UNN codons. MiaA-mediated changes in the proteome can in turn modify bacterial stress resistance, but varying levels of MiaA may also influence the availability of key metabolites like DMAPP that can have broad-ranging effects on multiple pathways. The MiaA-dependent phenotypes observed in this study with ExPEC parallel some of those associated with MiaA homologues in eukaryotes. For example, both MiaA and the human homologue TRIT1 can modulate the expression of electron transport chain complex subunits (120). Mutations in TRIT1 are associated with mitochondrial disease as well as the progression of some cancers (120–122). Only a limited number of tRNA substrates for TRIT1 have been identified in human cells (123). However, if TRIT1 acts akin to MiaA in ExPEC, it may also be able to directly and indirectly impact multiple regulatory factors and metabolites, either enhancing cellular health when TRIT1 activities are properly tuned or promoting disease when out of balance.

## DATA AVAILABILITY

The mass spectrometry proteomics data have been deposited to the ProteomeXchange Consortium via the PRIDE partner repository (124) with the dataset identifier PXD024782.

## Supplementary Material

gkac116_Supplemental_Files
