# Supplementary material for: A tRNA modifying enzyme as a tunable regulatory nexus for bacterial stress responses and virulence
Source: Nucleic Acids Res. 2022 Feb 25;50(13):7570–90. doi: 10.1093/nar/gkac116 (PMC9303304; doi:10.1093/nar/gkac116)
Supplement: gkac116_Supplemental_Files [file gkac116_supplemental_files.zip › 0202022_31_NAR_SuppInformation_MiaA.pdf]

SUPPLEMENTAL FIGURES

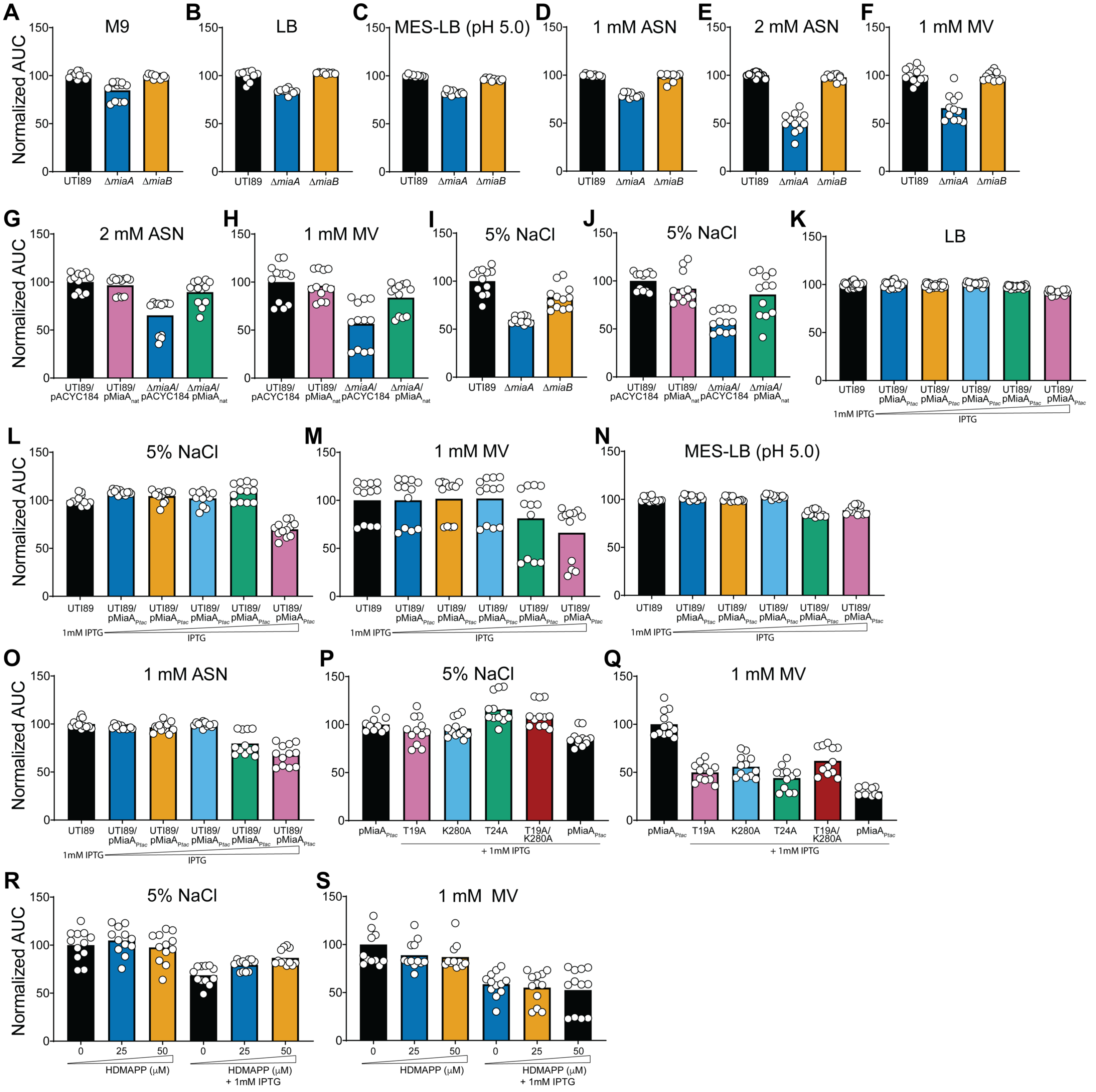

Supplemental Figure 1. Graphs display Area Under the Curve (AUC) data for all growth curves represented in Figures 2, 3, 5, and 8. The AUC graphs correspond with the main figures: (**A**) Figure 2A, (**B**) Figure 2B, (**C**) Figure 2C, (**D**) Figure 2D, (**E**) Figure 2E, (**F**) Figure 2F, (**G**) Figure 2G, (**H**) Figure 2H, (**I**) Figure 3C, (**J**) Figure 3D, (**K**) Figure 5B, (**L**) Figure 5C, (**M**) Figure 5D, (**N**) Figure 5E, (**O**) Figure 5F, (**P**) Figure 8B-i, (**Q**) Figure 8B-ii, (**R**) Figure 8C-i, and (**S**) Figure 8C-ii. Bars indicate mean values of 12 technical replicates (circles) from 3 independent experiments, normalized to control.

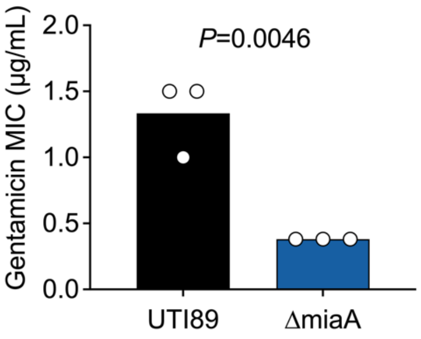

Supplemental Figure 2. UTI89Δ*miaA* is more sensitive to gentamicin than the wild-type UTI89. Bars in graph indicate mean MIC values (± SD) determined from three independent Etests. *P* value was determined by an unpaired Student's *t* test, n = 3.

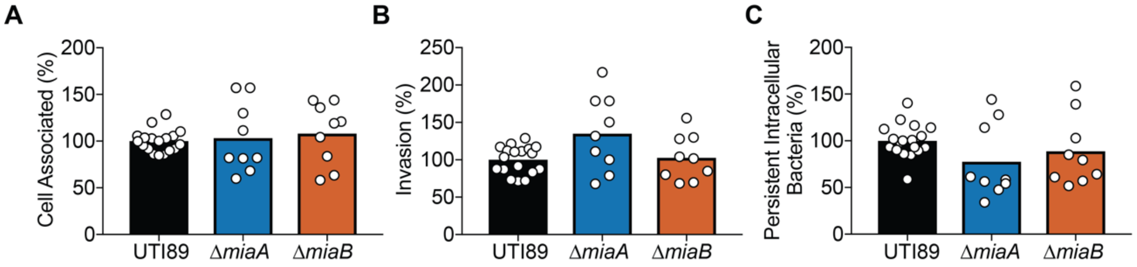

Supplemental Figure 3. MiaA and MiaB do not affect the ability of ExPEC to bind, invade, or persist intracellularly within bladder epithelial cells. Human bladder epithelial cells (5637 cells) were infected with UTI89, UTI89Δ*miaA*, or UTI89Δ*miaB* for 2 h, followed by a second 2-h incubation in the presence of the bactericidal, host cell-impermeable antibiotic gentamicin (100 μg/ml). Graphs show (**A**) the levels of host cell-associated bacteria prior to the addition of gentamicin, (**B**) and the relative numbers of intracellular bacteria recovered after the 2-h incubation in media containing gentamicin. (**C**) Longer-term bacterial persistence within the bladder cells was assessed by continued incubation of infected host cells for an additional 12 h with gentamicin. For the longer persistence assays, a submaximal concentration of gentamicin (10 μg/ml) was used to prevent extracellular growth of UPEC while limiting possible leaching of the antibiotic into the host cells. Data are expressed relative to wild-type UTI89, with bars indicating median values from 9 to 18 independent experiments performed in triplicate.

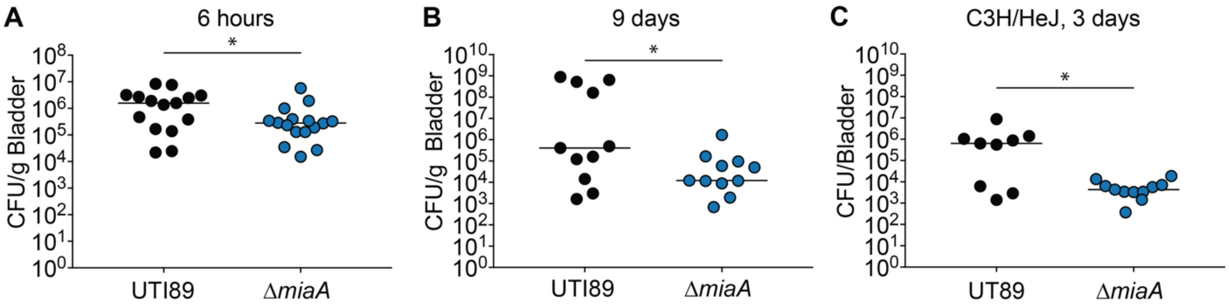

Supplemental Figure 4. MiaA promotes ExPEC colonization and persistence within the murine bladder. (**A and B**) The bladders of adult female CBA/J mice were inoculated via transurethral injections with ~10^7 CFU of wild-type UTI89 or UTI89Δ*miaA*. Mice were sacrificed (A) 6 hours or (B) 9 days later and bacterial titers within the bladders were determined by plating tissue homogenates. (**C**) Graph shows bacterial titers recovered from the bladders of adult female C3H/HeJ mice 3 days after inoculation with UTI89 or UTI89Δ*miaA*. Bars in all graphs denote median values. *, $P < 0.05$ by Mann Whitney U tests; $n \geq 9$ mice per group.

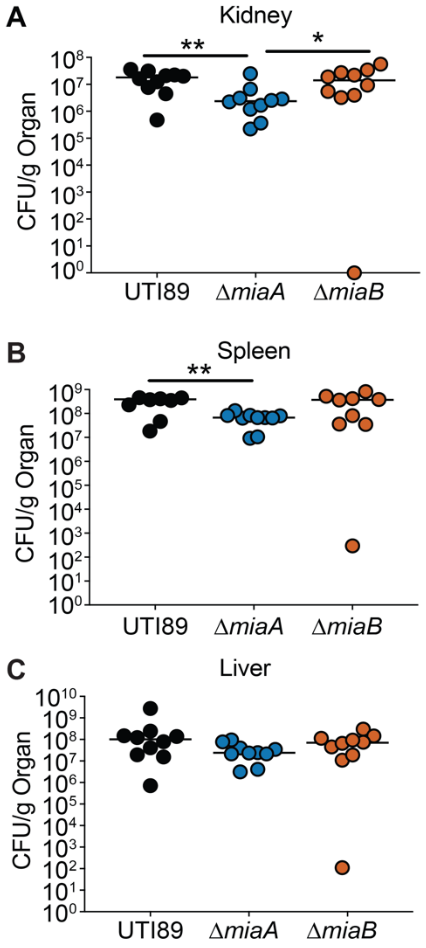

Supplemental Figure 5. MiaA promotes ExPEC fitness in a mouse model of sepsis. Adult female C57Bl/6 mice were inoculated via i.p. injections with 10^7-10^8 CFU of UTI89, UTI89Δ*miaA*, or UTI89Δ*miaB* and 6 hours later bacterial titers were present in the (**A**) kidneys, (**B**) spleen, and (**C**) liver were determined by plating tissue homogenates. *, $P < 0.05$ and **, $P > 0.01$ by Mann Whitney U tests; $n = 10$ mice per group.

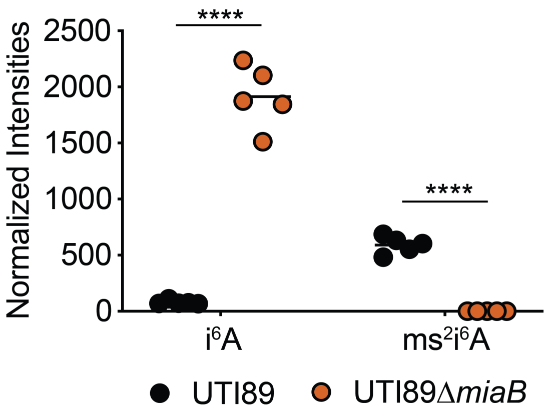

Supplemental Figure 6. The ms^2i^6A modification is missing in UTI89Δ*miaB*. RNA was collected from wild-type UTI89 and UTI89Δ*miaB* after reaching an OD_{600} of 0.5 in shaking LB cultures. Relative levels of i^6A and ms^2i^6A were determined by LC-MS. ****, $P < 0.0001$ as determined an unpaired *t* test; n = 5 independent replicates.

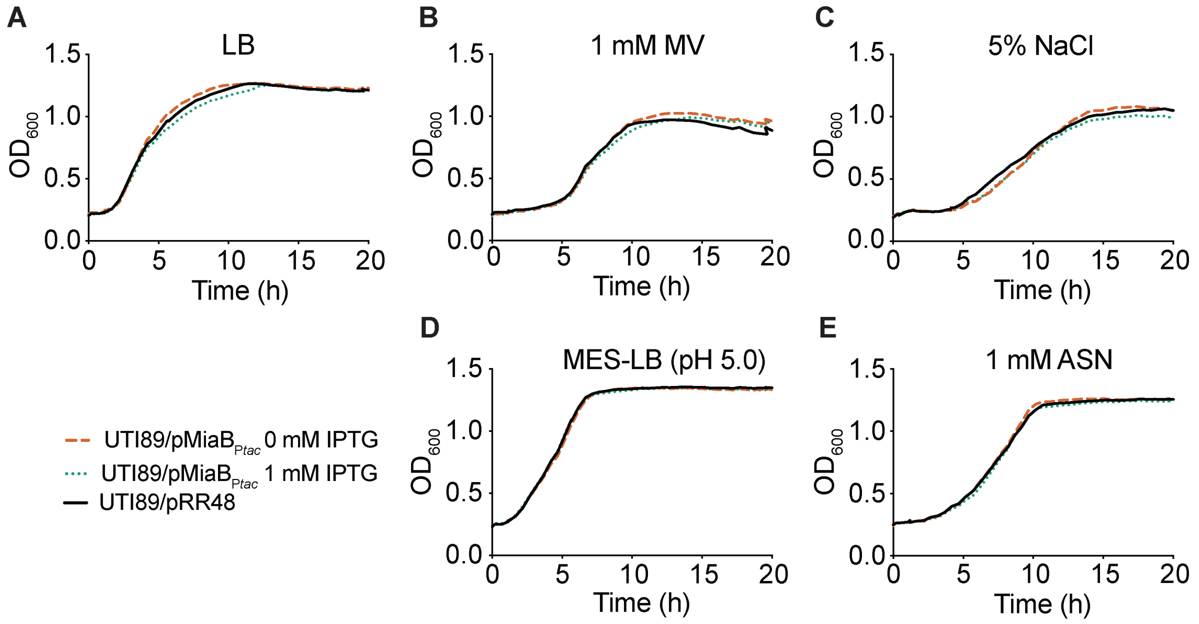

Supplemental Figure 7. Overproduction of MiaB does not affect growth of UTI89 under stressful conditions. Graphs show growth curves of UTI89 carrying pMiaB$_{Ptac}$ or the control plasmid pRR48 in (**A**) LB, (**B**) 1 mM MV, (**C**) 5% NaCl, (**D**) MES-LB, and (**E**) 1 mM ASN. To overexpress MiaB, 1 mM IPTG was added to UTI89/pMiaB$_{Ptac}$. Each curve indicates mean values from four replicates and are representative of three independent experiments.

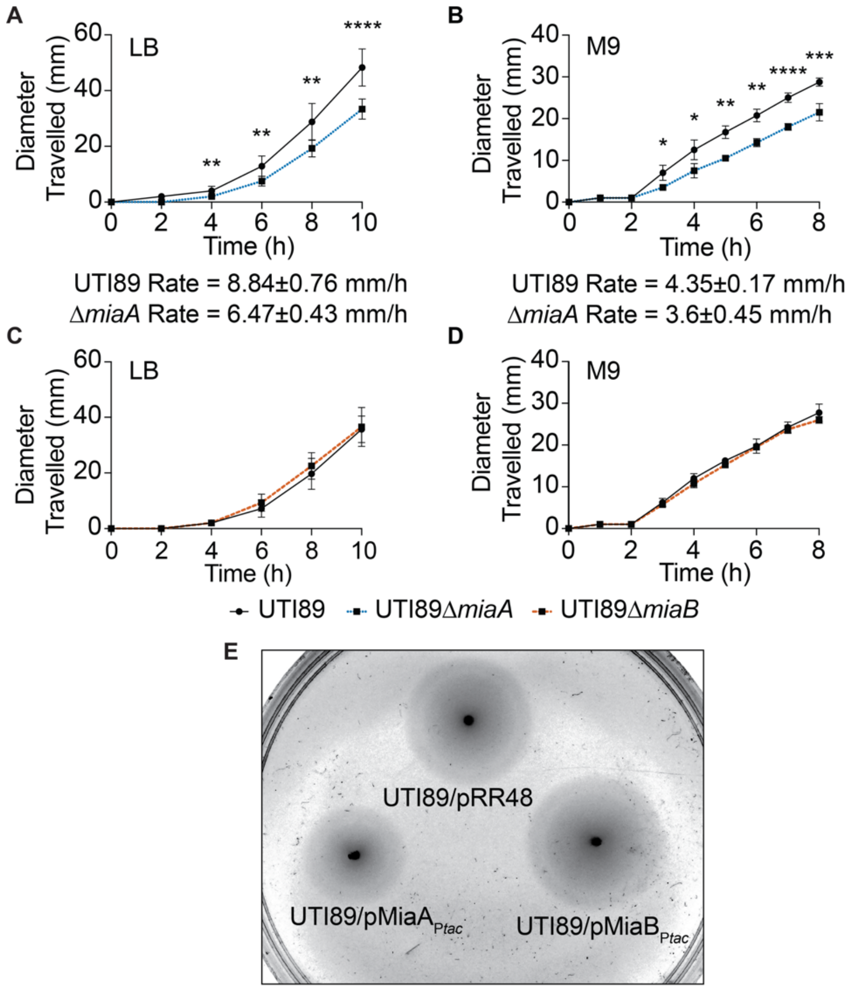

Supplemental Figure 8. MiaA modulates ExPEC motility. (**A - D**) Graphs indicate the spread of UTI89 (black lines), UTI89Δ*miaA* (dotted blue lines), and UTI89Δ*miaB* (dashed red lines) on (A and C) LB and (B and D) M9 swim motility plates incubated at 37°C. Shown are mean values ± SD from three independent experiments done in triplicate. Swim rates (± SD) for wild-type UTI89 and UTI89Δ*miaA* on LB and M9 swim plates are indicated below the graphs in (A) and (B). *, $P < 0.05$; **; $P < 0.01$; ***, $P < 0.001$; ****, $P < 0.0001$ versus wild-type UTI89, as determined by unpaired *t* tests; n ≥ 4 independent replicates. (**E**) Representative image showing the spread of UTI89/pRR48, UTI89/pMiaB$_{Ptac}$, and UTI89/pMiaA$_{Ptac}$ 6 hours after inoculation onto an LB swim plate containing 1 mM IPTG and ampicillin.

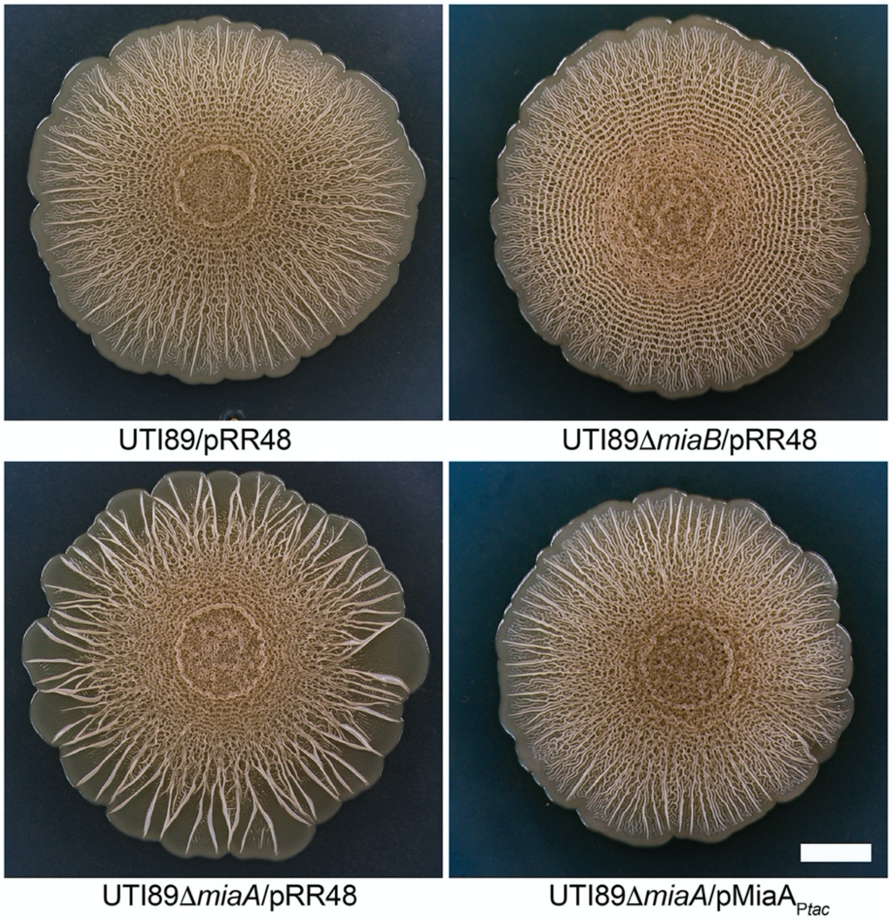

Supplemental Figure 9. MiaA regulates ExPEC biofilm development. Images show biofilms formed by wild-type UTI89 and its derivatives after 14 days of growth at room temperature on YESCA plates. Photos are representative of at least three independent replicates. Scale bar, 1 cm.

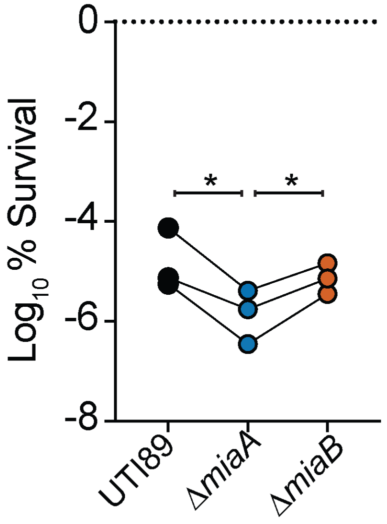

Supplemental Figure 10. UTI89Δ*miaA* has increased sensitivity to acid stress. After reaching mid-logarithmic growth phase in LB, wild-type UTI89, UTI89Δ*miaA*, and UTI89Δ*miaB* were exposed to acidic stress (pH 3.0) for 30 min. Following washes in PBS, surviving bacteria were enumerated by dilution plating. Titers are normalized to input. Biological replicates are connected by lines. *, $P < 0.05$ by paired t tests.

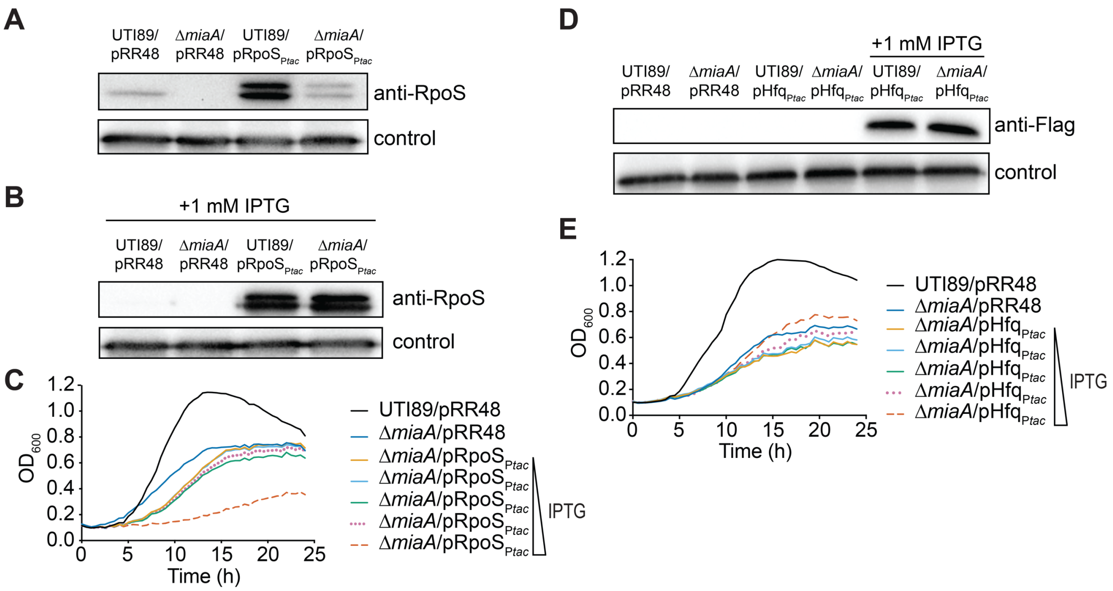

Supplemental Figure 11. Expression of RpoS or Hfq does not rescue growth of UTI89Δ*miaA* in the presence of high salt stress. (**A - C**) Western blots of RpoS and Flag-tagged Hfq in UTI89 and UTI89Δ*miaA* carrying pRpoS$_{Ptac}$, pHfq$_{Ptac}$, or the empty vector pRR48 following growth to stationary phase in LB or LB with 1 mM IPTG, as indicated. As a loading control, blots were also probed with anti-*E. coli* antibody. A shorter exposure was used for the blot shown in (B), making the RpoS band from UTI89/pRR48 notably lighter than the one shown in (A). Blots are representative of three independent experiments. (**D and E**) Curves show growth of the UTI89 and UTI89Δ*miaA* with the empty vector pRR48 or plasmids for IPTG-inducible expression of RpoS or Flag-tagged Hfq in LB + 5% NaCl. Cultures were grown shaking at 37°C with IPTG added in ten-fold increments from 0 to 1000 μM, as indicated. Each growth curve shows the means of results of four replicates from a single experiment and is representative of three independent experiments performed in quadruplicate.

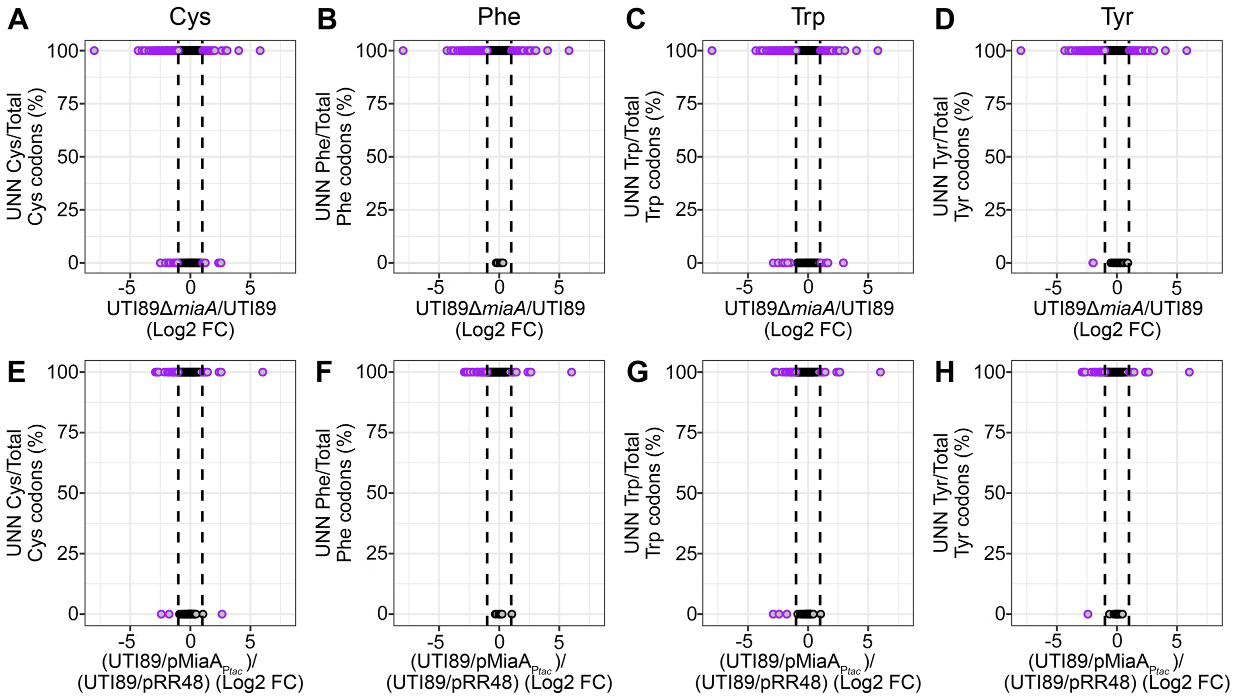

Supplemental Figure 12. UNN codon usage ratios for Cys, Phe, Trp, and Tyr correlate poorly with MiaA-sensitive protein expression. Plots show relative protein levels (Log2-fold change as determined by MudPIT**, see Figure 7**) versus UNN codon usage ratios for the indicated amino acids. Purple circles, proteins that were significantly changed (*P*<0.05, by Student's *t* tests) by at least 2-fold in UTI89Δ*miaA* or UTI89/pMiaA$_{Ptac}$ relative to their respective controls (*n* = 4 independent replicates for each group). Vertical dashed lines are placed at the 2-fold change cutoffs.

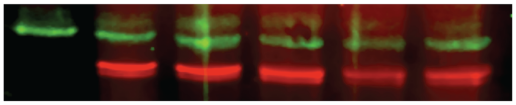

Supplemental Figure 13. Expression of Flag-tagged wild-type MiaA and MiaA point mutants. Immunoblot shows levels of Flag-tagged MiaA (red) recovered from recombinant UTI89 strains expressing wild-type (WT) MiaA or the indicated mutant MiaA variants. Bacteria were diluted 1:100 from overnight shaking cultures and grown for 2.5 h at 37ºC in LB prior to addition of IPTG (1 mM). After an additional hour incubation, bacteria were collected and processed for western blot analysis. EV, pRR48 empty vector control. A non-specific band that was detected using anti-*E. coli* antibody (green) serves as a loading control.

Supplemental Table 1. Bacterial strains and plasmids

Strain or Plasmid	Description	Source or Reference
**Strains**		
UTI89	UPEC reference strain, cystitis isolate	(1,2)
UTI89::Kan^R	UTI89 with a Kan^R resistance cassette inserted at the *att*Tn7 site	This study
UTI89Δ*miaA*	UTI89 *miaA::*Cam^R	This study
UTI89Δ*miaB*	UTI89 *miaB::*Cam^R	This study
**Plasmids**		
pACYC184	Low-copy number plasmid; Tet^R, Cam^R	New England Biolabs
pBAD18	Arabinose-inducible bacterial expression plasmid; Amp^R	(3)
pBAD33	Arabinose-inducible bacterial expression plasmid; Cam^R	(3)
pKD3	Carries FRT-flanked Cam^R cassette; template for use in lambda-Red-mediated recombination	(4)
pKD4	Carries FRT-flanked Kan^R cassette; template for use in lambda-Red-mediated recombination	(4)
pKM208	IPTG-inducible lambda Red recombinase expression plasmid; Amp^R	(5)
pRR48	Contains IPTG-inducible P_{tac} promoter upstream of the MCS; Amp^R	(6)
$pHfq_{Ptac}$	*hfq* from UTI89 cloned with C-terminal Flag and 6xHis tags into PstI, HindIII sites of pRR48; Amp^R	This Study
$pRpoS_{Ptac}$	*RpoS* cloned from UTI89 into PstI, HindIII sites of pRR48; Amp^R	(7)
$pMiaA_{Ptac}$	*miaA* cloned from UTI89 into PstI, KpnI sites of pRR48; Amp^R	This study
pMiaA-$Flag_{Ptac}$	*miaA* with C-terminal Flag tag sequences sub-cloned from $pMiaA_{nat}$ into the PstI and KpnI sites of pRR48; Amp^R	This study
$pMiaB_{Ptac}$	*miaB* cloned from UTI89 into PstI, KpnI sites of pRR48; Amp^R	This study
$pMiaA_{nat}$	*miaA* plus 200 bp of flanking sequences cloned from UTI89 into the EcoR1 site of pACYC184; Tet^R	This study
pMiaA-$Flag_{nat}$	pACYC184-derived plasmid encoding MiaA with C-terminal Flag tag plus linker under control of native *miaA* promoter; Tet^R	This study

p2Luc-HIV	Eukaryotic reporter construct with the HIV *gag-pol* frameshift region inserted between the renilla and firefly luciferase genes. The HIV linker sequence contains a 2-nucleotide insertion resulting in a stop codon located 6 codons after the start of firefly luciferase gene. The firefly gene is in a -1 frame relative to the upstream renilla gene; Amp^R	Derived from (8)
p2Luc-HIV-IF	Control for p2Luc-HIV. The HIV *gag-pol* linker was altered to keep the renilla and firefly luciferases in-frame; Amp^R.	Derived from (8)
p2Lucaz1	Eukaryotic reporter construct with the Az1 frameshift region inserted between the renilla and firefly luciferase genes. The Az1 linker sequence contains a stop codon positioned in-frame so that a +1 frameshift must occur for read-through expression of firefly luciferase; Amp^R	(9)
p2Lucaz1-IF	Control for p2Lucaz1. The Az linker was altered to keep the renilla and firefly luciferases in-frame; Amp^R	(9)
pCWR42-CamR	Dual luciferase reporter with in-frame Az1 linker cloned from p2Lucaz1-IF into pBAD33. Has a Shine-Dalgarno sequence and is under control of the arabinose-inducible P_{BAD} promoter. Control for pCWR43-CamR; Cam^R	This study
pCWR42-AmpR	Dual luciferase reporter with in-frame Az1 linker cloned from p2Lucaz1-IF into pBAD18. Has a Shine-Dalgarno sequence and is under control of the arabinose-inducible P_{BAD} promoter. Control for pCWR43-AmpR; Amp^R	This study
pCWR43-CamR	Dual luciferase reporter with Az1 linker cloned from p2Lucaz1 into pBAD33. Has a Shine-Dalgarno sequence and is under control of the arabinose-inducible P_{BAD} promoter; Cam^R	This study
pCWR43-AmpR	Dual luciferase reporter with Az1 linker cloned from p2Lucaz1 into pBAD18. Has a Shine-Dalgarno sequence and is under control of the arabinose-inducible P_{BAD} promoter; Amp^R	This study
pCWR44-CamR	Dual luciferase reporter with in-frame HIV linker cloned from p2Luc-HIV-IF into pBAD33. Has a Shine-Dalgarno sequence and is under control of the arabinose-inducible P_{BAD} promoter. Control for pCWR45-Cam; Cam^R	This study
pCWR44-AmpR	Dual luciferase reporter with in-frame HIV linker cloned from p2Luc-HIV-IF into pBAD18. Has a Shine-Dalgarno sequence and is under control of the arabinose-inducible P_{BAD} promoter. Control for pCWR45-Amp; Amp^R	This study
pCWR45-CamR	Dual luciferase reporter with HIV linker cloned from p2Luc-HIV into pBAD33. Has a Shine-	This study

	Dalgarno sequence and is under control of the arabinose-inducible P_{BAD} promoter; Cam^R	
pCWR45-AmpR	Dual luciferase reporter with HIV linker cloned from p2Luc-HIV into pBAD18. Has a Shine-Dalgarno sequence and is under control of the arabinose-inducible P_{BAD} promoter; Cam^R	This study
pMiaA(T19A)$_{Ptac}$	T19A mutation in MiaA generated using QuikChange kit with pMiaA-Flag$_{Ptac}$ as template; Amp^R	This study
pMiaA(K280A)$_{Ptac}$	K280A mutation in MiaA generated using QuikChange kit with pMiaA-Flag$_{Ptac}$ as template; Amp^R	This study
pMiaA(T24A)$_{Ptac}$	T24A mutation in MiaA generated using QuikChange kit with pMiaA-Flag$_{Ptac}$ as template; Amp^R	This study
pMiaA(T19A/K280A)$_{Ptac}$	T19A mutation introduced using QuikChange kit with pMiaA(K280A)$_{Ptac}$ as template; Amp^R	This study

Supplemental Table 2. Primers used in this study

Primer Name[a]	Sequence (5’-3’)[b]
Cloning primers	
MiaA-pRR48-F	CGCGCTGCAGATGAGTGATATCAGTAAGGCG
MiaA-pRR48-R	CGGCGGTACCTCAGCCTGCGATAGCACCAAC
MiaA-Flag-pRR48-F	CAGACCTGCAGATGAGTGATATCAGTAAGGCGAGCC
MiaA-Flag-pRR48-R	CAGACGGTACCCTATCCCTTATCGTCGTCATCCTTGT
MiaB-pRR48-F	CGCGCTGCAGATGACCAAAAAACTCCATATTAAAACC
MiaB-pRR48-R	CGGCGGTACCGAATTACGGCTGATAATAAC
MiaA-Flag-pACYC184-F	CGGCGAATTCGGCTAAAAGTTTCTGGCGAAGAAAAATCGG
MiaA-Flag-pACYC184-R	CGCGGAATTCCTATCCCTTATCGTCGTCATCCTTGTAGTCTGGTC CTCCTCCTCCGCCTGCGATAGCACCAACAAC
MiaA-pACYC184-F	CGCGGAATTCGCCCCTTAGCCATTCTCTCTTTTCCTTATATG
MiaA-pACYC184-R	CGGCGAATTCCCAGTCCGATGCGCAGCATGTGACCATC
Hfq-pRR48-PstI	CATACCTGCAGATGGCTAAGGGGCAATCTT
Hfq-CFLAG-his-HindII	CATACAAGCTTCTAGTGGTGGTGGTGGTGGTGTCCCTTATCGTCG TCATCCTTGTAGTCTCC TTCGGTTTCTTCGCTGTCCT
p2Luc-F	GCCGGGGTACCAGGAGGTCAGTCAGATGACTTCGAAAGTTTATG ATCCAG
p2Luc-R	GCCGGAAGCTTTTACAATTTGGACTTTCCGCCC
QuickChange primers	
MiaA-Flag_K280A-F	CACGAGACAGTTGGCGGCGCGGCAGATAACCTGG
MiaA-Flag_K280A-R	CCAGGTTATCTGCCGCGCCGCCAACTGTCTCGTG
MiaA-Flag_T19A-F	TGATGGGGCCGGCGGCCTCCGGT
MiaA-Flag_T19A-R	ACCGGAGGCCGCCGGCCCCATCA
MiaA-Flag_T24A-F	CGACGGCCTCCGGTAAAGCAGCGTTAGCC
MiaA-Flag_T24A-R	GGCTAACGCTGCTTTACCGGAGGCCGTCG
Knockout, insertion, and confirmation primers	
*att*Tn7KanR-KI-F	TCTGGCGTAGCCTGGGAGTTATTGCCGGATGCGATGCTGGTGTGT AGGCTGGAGCTGCTTCG
*att*Tn7KanR-KI-R	TCACGTAAAAAAACGTCTAATCCGTAGACCGGATAAGAGGCATATG AATATCCTCCTTAG
MiaA-KO-F	CGATAAAAGCCCTGAAAGATGAGTGATATCAGTAAGGCTGTGTAGG CTGGAGCTGCTTCG
MiaA-KO-R	CGTCTCCTGACGTTTGCGTCAGTTCCGTTAAAGTTTTACCCATATGA ATATCCTCCTTAG
MiaA-KO-Conf-F	GCCGCCGGGTGGTCTGTTAC
MiaA-KO-Conf-R	CAGCCTGCGATAGCACCAAC
MiaB-KO-F	CCTGCATTCCTGGCTACTATTTCGCAAGAGCAAGTCGTGTGTAGGC TGGAGCTGCTTCG
MiaB-KO-R	CGGCGGGCCTGAGAATTACGGCTGATAATAACCCACGCCATATGA ATATCCTCCTTAG
MiaB-KO-Conf-F	GCCGACCATTCCTCCGCCGAC
MiaB-KO-Conf-R	CATTGTCTGCTGGCTCCAGG

RT-qPCR primers	
miaA-F	TACGGACTTGCCTTCCATTC
miaA-R	GCGCAAACACCTCGATAAAC
miaB-F	GTAGAAGGTACATCGCGTAAG
miaB-R	TCGGGTAGACGTCGGTAAT
rpoD-F	TTCGTACGCAAGAACGTCTG
rpoD-R	AGGTATCGCTGGTTTCGTTG

[a]F, forward primer; R, reverse primer; KO, knockout primer; KI, knock-in primer; Conf, confirmation primer. [b]Added restriction sites in cloning primers underlined.

Supplemental Table 3. Correlations between protein abundance and UNN content

	**UTI89Δ*miaA*/UTI89**		**(UTI89/pMiaA$_{Ptac}$)/(UTI89/pRR48)**	
**Consecutive UNNs***	**rho**	***P* value**	**rho**	***P* value**
**1**	-0.17	3.42E-10	-0.20	7.18E-14
**2**	-0.16	6.52E-10	-0.20	7.18E-14
**3**	-0.09	1.46E-03	-0.10	1.62E-04
**4**	-0.04	1.65E-01	-0.06	3.47E-02
**5**	-0.02	4.78E-01	-0.06	2.09E-02
**6**	0.04	1.21E-01	-0.03	2.68E-01
**Total UNN codons****	-0.08	2.47E-03	-0.21	2.91E-16

Two-sided Spearman's rank correlation coefficients (rho) and FDR-corrected *P* values (Benjamini-Hochberg) were used to assess relationships between UNN values and Log2-fold change ratios of protein abundance, as determined by MudPIT analysis (see **Supplemental Dataset 1).**
*When determining consecutive UNN values, each UNN within an open reading frame was counted only once (e.g. UNN codons that were within a triplicate UNN sequence were not also counted as singlets or duplicates). Values calculated using R, version 4.0.5.
**Considers all UNN codons within an open reading frame, regardless of location relative to other UNN codons.

Supplemental Dataset 1 (separate file). Excel file with results from MudPIT analysis of proteins expressed by UTI89, UTI89Δ*miaA*, UTI89/pRR48, and UTI89/pMiaAP*tac* grown in LB to mid-log phase. The file contains 15 worksheets, with the first containing a description of all worksheet contents. Proteins that are differentially expressed due to *miaA* deletion or overexpression were assigned to one or more of 14 functional categories (see *Categories* worksheet and embedded graph). Codon frequencies (*UNN Stats* worksheet) were calculated for each gene in UTI89 (accessions CP000243.1 and CP000244.1) using custom Python scripts that leverage the BioPython and NumPy packages.

SUPPLEMENTAL REFERENCES

1. Mulvey, M.A., Schilling, J.D. and Hultgren, S.J. (2001) Establishment of a persistent *Escherichia coli* reservoir during the acute phase of a bladder infection. *Infect Immun*, **69**, 4572-4579.
2. Chen, S.L., Hung, C.S., Xu, J., Reigstad, C.S., Magrini, V., Sabo, A., Blasiar, D., Bieri, T., Meyer, R.R., Ozersky, P. *et al.* (2006) Identification of genes subject to positive selection in uropathogenic strains of *Escherichia coli*: a comparative genomics approach. *Proc Natl Acad Sci U S A*, **103**, 5977-5982.
3. Guzman, L.M., Belin, D., Carson, M.J. and Beckwith, J. (1995) Tight regulation, modulation, and high-level expression by vectors containing the arabinose PBAD promoter. *J Bacteriol*, **177**, 4121-4130.
4. Datsenko, K.A. and Wanner, B.L. (2000) One-step inactivation of chromosomal genes in *Escherichia coli* K-12 using PCR products. *Proc Natl Acad Sci U S A*, **97**, 6640-6645.
5. Murphy, K.C. and Campellone, K.G. (2003) Lambda Red-mediated recombinogenic engineering of enterohemorrhagic and enteropathogenic *E. coli*. *BMC Mol Biol*, **4**, 11.
6. Zhou, Q., Ames, P. and Parkinson, J.S. (2009) Mutational analyses of HAMP helices suggest a dynamic bundle model of input-output signalling in chemoreceptors. *Mol Microbiol*, **73**, 801-814.
7. Donovan, G.T., Norton, J.P., Bower, J.M. and Mulvey, M.A. (2013) Adenylate cyclase and the cyclic AMP receptor protein modulate stress resistance and virulence capacity of uropathogenic *Escherichia coli*. *Infect Immun*, **81**, 249-258.
8. Grentzmann, G., Ingram, J.A., Kelly, P.J., Gesteland, R.F. and Atkins, J.F. (1998) A dual-luciferase reporter system for studying recoding signals. *RNA (New York, N.Y.)*, **4**, 479-486.
9. Howard, M.T., Shirts, B.H., Zhou, J., Carlson, C.L., Matsufuji, S., Gesteland, R.F., Weeks, R.S. and Atkins, J.F. (2001) Cell culture analysis of the regulatory frameshift event required for the expression of mammalian antizymes. *Genes Cells*, **6**, 931-941.
